# Effect of Pharmacist Email Alerts on Concurrent Prescribing of Opioids and Benzodiazepines by Prescribers and Primary Care Managers: A Randomized Clinical Trial

**DOI:** 10.1001/jamahealthforum.2022.3378

**Published:** 2022-09-30

**Authors:** Adam Sacarny, Elana Safran, Mary Steffel, Jacob R. Dunham, Orolo D. Abili, Lobat Mohajeri, Patricia T. Oh, Alan Sim, Robert E. Brutcher, Christopher Spevak

**Affiliations:** 1 Department of Health Policy and Management, Mailman School of Public Health, Columbia University, New York, New York; 2 Office of Evaluation Sciences, US General Services Administration, Washington, DC; 3 D’Amore-McKim School of Business, Northeastern University, Boston, Massachusetts; 4 Vista Defense Technologies, Rock Island, Illinois; 5 Department of Anesthesia, Walter Reed National Military Medical Center, Bethesda, Maryland; 6 Department of Pharmacy, Walter Reed National Military Medical Center, Bethesda, Maryland; 7 Enterprise Intelligence and Data Solutions Program Management Office, Defense Health Agency, Falls Church, Virginia; 8 School of Medicine, Uniformed Services University of the Health Sciences, Bethesda, Maryland; 9 National Capital Regional Pain Initiative, Department of Anesthesia and Pain Medicine, Walter Reed National Military Medical Center, Bethesda, Maryland

## Abstract

**Importance:**

Policy makers have sought to discourage concurrent prescribing of opioids and benzodiazepines (coprescribing) because it is associated with overdose. Email alerts sent by pharmacists may reduce coprescribing, but this intervention lacks randomized evidence.

**Objective:**

To investigate whether pharmacist emails to practitioners caring for patients who recently received opioids and benzodiazepines reduce coprescribing of these medications.

**Design, Setting, and Participants:**

Randomized clinical trial (intention to treat) conducted in 2019-2021 of patients and their practitioners (prescribers and primary care managers) in the National Capital Region of the Military Health System. Participants were 2237 patients who were recently coprescribed opioids and benzodiazepines. These patients had 789 practitioners eligible for emails.

**Interventions:**

Patients were randomized to email alerts to their practitioners or as-usual care. Clinical pharmacists sent the email alert. Messages were standardized and designed to facilitate coordination between practitioners, increase awareness of guidelines, and provide action steps and resources.

**Main Outcomes and Measures:**

The primary outcomes were patients’ days received of opioids, benzodiazepines, and concurrent opioids and benzodiazepines during the 90 days following enrollment evaluated using 1-sided hypothesis tests. Secondary outcomes included total prescribing of opioids and benzodiazepines by patients’ practitioners, including to patients outside the study, to test for broader outcomes on their prescribing.

**Results:**

Of 2237 patients, 1187 were assigned to treatment and 1050 to control; 1275 (57%) were women. Patients received a mean (SD) of 31 (44) days of opioids and 33 (34) days of benzodiazepines in the 90 days before enrollment. There were no detected differences in the primary end points, including patients’ receipt of opioids (adjusted difference, 1.1 days; 95% CI, -∞ to 3.0; *P* = .81), benzodiazepines (adjusted difference, −0.6 days; 95% CI, -∞ to 1.4; *P* = .30), and opioids and benzodiazepines together (adjusted difference, −0.1 days; 95% CI, -∞ to 0.7; *P* = .41). Of 789 practitioners, 429 were considered the treatment group, 325 were considered controls, and 35 were excluded. There were no detected differences in practitioners’ total prescribing of opioids, benzodiazepines, or both drug classes together.

**Conclusions and Relevance:**

In this randomized clinical trial of pharmacist emails to practitioners, email alerts failed to detectably reduce coprescribing, highlighting the value of alternative approaches. Combining randomization with quality improvement activities may help stakeholders seeking evidence-based interventions to encourage guideline-concordant care.

**Trial Registration:**

ClinicalTrials.gov Identifier: NCT03887247

## Key Points

**Question:**

Can pharmacist email alerts to practitioners reduce concurrent prescribing of opioids and benzodiazepines?

**Findings:**

In this randomized clinical trial of 2237 patients coprescribed opioids and benzodiazepines and 789 practitioners who treated them, email alerts failed to detectably reduce concurrent prescribing of opioids and benzodiazepines, which can put patients at risk of overdose. The email alerts had no statistically significant effect on patient receipt of these medications or on practitioner prescribing.

**Meaning:**

These findings suggest that alternative strategies may be more fruitful targets for efforts to make opioid prescribing safer and demonstrate that pairing randomization with quality improvement activities can generate evidence for stakeholders.

## Teaser

This randomized clinical trial investigates whether pharmacist emails to practitioners of patients who recently received opioids and benzodiazepines reduce coprescribing of these medications.

## Introduction

During the past 2 decades, opioid overdoses and deaths have increased substantially in what is widely described as a public health crisis.^1,2,3,4^ Harms from benzodiazepines have followed a similar trajectory but have attracted less attention.^5,6,7^ These medications heighten opioid-induced respiratory depression, the cause of opioid overdose.^8^ Concurrent receipt of prescribed opioids and benzodiazepines is associated with adverse patient outcomes.^9,10,11^ One-third to one-half of prescription opioid overdose deaths involve a benzodiazepine.^12,13^ In 2017, more than 1 in 5 patients prescribed an opioid also received a benzodiazepine.^14,15^ While this rate has declined in recent years, 3 million adults still receive concurrent prescriptions (coprescriptions) annually.^16^

These developments have led policy makers to discourage coprescribing of these medications. Recommendations to avoid coprescribing appear in guidelines from the Centers for Disease Control and Prevention and the Department of Veterans Affairs and the Department of Defense,^17,18^ Choosing Wisely guidance from the American Society of Anesthesiologists,^19^ and the Beers Criteria from the American Geriatrics Society.^20^ The US Food and Drug Administration also requires black box warnings about overdose on all opioid and benzodiazepine product labeling.^21^

The ongoing receipt of opioids and benzodiazepines together highlights the need for evidence-based approaches to encourage safer prescribing. Nudges, or interventions that seek to change behavior without directly limiting choices or changing incentives, provide one approach.^22,23^ There are several examples of successful opioid prescribing nudges, including peer comparison feedback on pills per opioid prescription,^24^ reduced default duration or quantity for new opioid prescriptions,^25,26,27,28^ and letters to practitioners informing them that one of their patients overdosed.^29^ Nudgelike interventions have also successfully reduced benzodiazepine prescribing.^30,31^ Nonrandomized studies of engaging pharmacists to deliver interventions to the rest of the care team have reported the interventions as effective strategies,^32,33,34^ as have clinical trials with pharmacists as participants in the intervention.^31,35^ Yet there is little randomized evidence on using nudges to decrease opioid-benzodiazepine coprescribing. Evidence is also lacking on whether including pharmacists in efforts to reduce coprescribing could make them more successful.

We therefore conducted a randomized clinical trial (intent to treat) of email alerts from clinical pharmacists to practitioners after their patients filled coprescriptions of opioids and benzodiazepines. If these messages reduced subsequent prescribing, they could be valuable tools for policy makers, organizations, and practitioners seeking to make health care delivery safer.

## Methods

### Study Design and Participants

The trial used a parallel-group design in which patients were randomized weekly to the treatment (email alert to their practitioners) or control (no alert) arms. The allocation ratio was 1:1 except for the first 421 patients, when we were testing an additional similar email alert. Further information appears in the Trial Protocol included as Supplement 1. At this time, the ratio was 1:1:1 to the 2 alert arms or control, or 2:1 to either alert vs control.

The intervention was considered quality improvement (QI) by the Walter Reed National Military Medical Center institutional review board. The evaluation was overseen by this institutional review board as human participant research and was exempt from informed consent requirements. This study follows the Consolidated Standards of Reporting Trials (CONSORT) reporting guideline.

Study participants were patients in the National Capital Region (NCR) of the Military Health System and their associated NCR practitioners, defined as their opioid prescribers, benzodiazepine prescribers, and primary care manager. Patients who had recently filled overlapping opioid and benzodiazepine prescriptions were identified weekly in the Military Health System Population Health Portal Opioid Management Registry, which provides near real-time data on opioid prescribing in the Military Health System. Prescriptions written by non-NCR practitioners and community practitioners were counted when enrolling patients. However, only NCR practitioners were eligible for emails. Only primary care managers with a prior care relationship with the patient, defined as having at least 1 appointment in the past year, were eligible for emails.

To meet study inclusion criteria, the patient needed at least 1 opioid-benzodiazepine overlap day in the last month according to the dates of service and days’ supply of their prescription drug fills. We also required the patient have at least 1 practitioner eligible for emails (for the first 421 patients, we required at least 2 practitioners). Patients receiving hospice care, with a cancer diagnosis, or under age 18 years were excluded from enrollment, as were those who had already been enrolled. We capped weekly enrollment to limit pharmacist workload, with patients with more recent overlaps enrolled first.

Calculations from pre-intervention data indicated that a sample of 2500 patients would yield 80% statistical power to detect reductions in opioid prescribing of 2.0 days, benzodiazepine prescribing of 1.9 days, and concurrent prescribing of 1.1 days at the 5% significance level using 1-sided tests.

### Intervention

The intervention was an email alert to the patient’s practitioners. The message (available in the Trial Protocol Supplement 1) informed the practitioners that their patient may be at risk for a life-threatening opioid-benzodiazepine interaction. It encouraged them to take immediate action and, when multiple practitioners were identified, to coordinate with each other to revise the patient’s treatment plan. It sought to facilitate this coordination by allowing practitioners to reply all to the group and by listing practitioners’ phone numbers. The message noted the Veterans Affairs/Department of Defense clinical guideline and risk to patient, action steps including tapering and prescribing naloxone, and relevant resources about how to safely modify the patient’s treatment plan. It also included a report of the patient’s recent opioid and benzodiazepine prescriptions and information on risk factors and recent health care use related to overdose risk.

Initially, we also tested a version of the alert in which emails were sent to each practitioner individually and did not include practitioners’ contact information. Given their similarity, we do not distinguish between the 2 alerts in analyses and consider the 140 patients assigned to this alert treated.

Clinical pharmacists sent the message to practitioners as an encrypted email with the report attached. Enrollment occurred weekly from June 2019 to March 2021 except weeks when pharmacists were on vacation (8 weeks) or computer systems were undergoing maintenance (16 weeks). The prespecified analysis plan was finalized in January 2021. Researchers were unblinded to study data after enrollment ended in March 2021.

### Randomization

Patients were allocated to treatment and control arms each week using a random sequence of numbers. Randomization was stratified by ordering patients by their volume of opioid-benzodiazepine overlap during the previous 90 days, measured in days. We then divided the sorted list into blocks of 3 (first 421 patients) or 2 (subsequent patients). The enrollment list was sent to the clinical pharmacist team along with a packet of prepopulated emails and reports. The pharmacists then emailed the patients’ practitioners.

### Data Sources

Our analyses use 2019-2021 prescription fill and military enrollment records reported in the Military Health System Management and Reporting Tool database. We observe fills at military pharmacies as well as community pharmacies covered by TRICARE, the military health insurance benefit. Analyses were conducted in Stata/MP version 17 (StataCorp).

### Primary and Secondary End Points

The primary analysis was conducted at the patient level. We prespecified 3 primary end points owing to the letter’s focus on opioids, benzodiazepines, and overlap thereof. The first was the patient’s receipt of opioids measured as the total days’ supply of all opioid prescription drug fills during the 90 days following enrollment. The second end point was the patient’s receipt of benzodiazepines measured analogously. The final end point was the number of days the patient received overlapping opioids and benzodiazepines during the 90-day period. To measure overlap, we assumed a patient’s opioid or benzodiazepine fill became active on its fill date and lasted for its days’ supply.

Secondary patient-level end points included measuring the primary end points at shorter and longer durations; measuring these end points as the number of fills or the morphine or diazepam milligram equivalents; and tracking days’ supply of naloxone, substance use disorder treatment drugs, and other psychoactive and analgesic medications.

To assess effects of the intervention on practitioners, including changes in prescribing to their other patients who were not mentioned in the email alerts, we prespecified secondary analyses at the practitioner level capturing their prescribing to all patients. We assembled cohorts of treatment and control group practitioners. The cohorts consisted of the opioid prescribers, benzodiazepine prescribers, and/or primary care managers of study patients. We grouped practitioners into treatment or control based on the randomized treatment assignment of their first patient enrolled in the study. We considered the date a practitioner’s first patient was enrolled in the study as the practitioner’s enrollment date for outcome measurement. Practitioners in the control group could later receive emails if their patients were enrolled in subsequent weeks. For this reason, we consider the practitioner-level evaluation an imperfect compliance trial yielding intention-to-treat estimates.

Analyses at the practitioner level consisted of 3 end points. The first was prescribing of opioids to all patients as measured by the total days’ supply of all fills prescribed by the practitioner and dispensed during the 90 days following enrollment. The second was prescribing of benzodiazepines measured analogously. The last was the number of opioid-benzodiazepine overlap days to which the practitioner contributed during the 90-day postenrollment period. We calculated this end point by assuming a patient started taking a prescription on the fill date and continued for its days’ supply. For each patient receiving opioids and/or benzodiazepines from the practitioner, we counted the number of days during the 90-day period when they were taking medications in both classes and the practitioner prescribed at least one of them.

### Statistical Analysis

Patient-level analyses use multivariable linear regression models with inverse probability of treatment weighting to account for differing allocation ratios early vs late in the study period. All regressions adjust for opioid days, benzodiazepine days, and overlap days during the 90-day period immediately prior to enrollment. Other secondary end points are additionally adjusted for the end point as measured during the 90-day pre-enrollment period. Regressions include fixed effects for randomization strata. Practitioner-level analyses use the same models, assigning each practitioner the weighting of their first enrolled patient and including fixed effects for first enrollment week rather than randomization strata.

Analyses use robust variance estimates for inference. Practitioner-level analyses are clustered at the level of the first enrolled patient. We hypothesized that the intervention would lower prescribing and that, regardless, it was highly unlikely to increase it. Therefore, for each primary end point, we conducted a 1-sided hypothesis test with the alternative hypothesis that the effect was negative (implying a reduction in prescribing) and considered *P* < .05 significant. To address the multiplicity of testing, we also conducted hypothesis tests that effects on the 3 primary end points were jointly 0 and reported the single 1-sided *P* value.^36^ We use the same approach for the 3 analogous practitioner-level outcomes. All other secondary end points are treated as exploratory, use 2-sided tests with *P* < .05 considered significant, and do not adjust for multiple testing.

## Results

**Figure 1.**
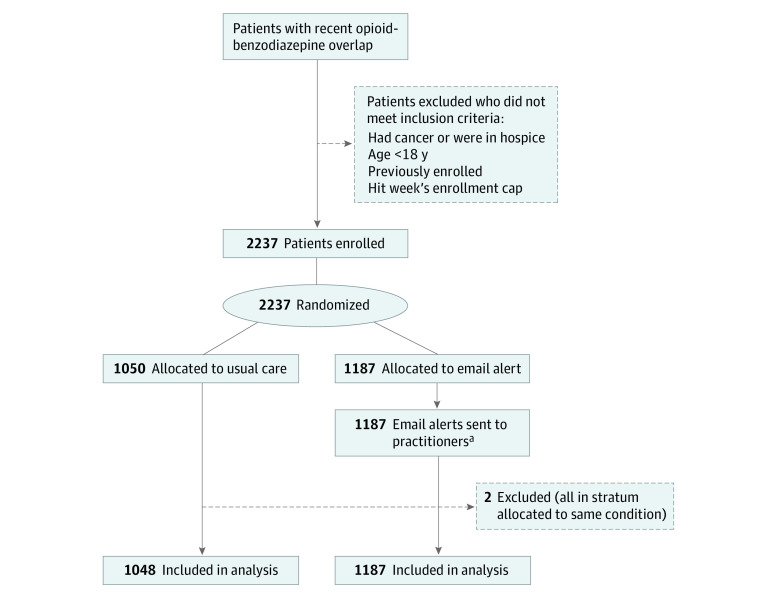
Flow Diagram of Patients in Study ^a^Includes the small number of patients with practitioners who could not be contacted. Among 1989 patient-practitioner pairs in the email alert group, 50 (2.5%) had email addresses that could not be resolved. For these patients, pharmacists sent the email to the patient’s remaining practitioners with valid addresses. If there were no remaining practitioners, the email was not sent.

A total of 2237 patients were enrolled in the study, with 1050 allocated to the control group and 1187 allocated to the treatment group (Figure 1). After exclusions, 2235 patients remained in the analysis sample. The mean (SD) number of contactable practitioners per patient was 1.6 (0.8) (Table 1; eTable 1 in Supplement 2). Patients received a mean (SD) of 31 (44) days of opioids and 33 (34) days of benzodiazepines during the 90 days before enrollment with 9 (15) days of opioid-benzodiazepine overlap during that time. More than half of patients had a mental health disorder diagnosis prior to enrollment and 1 in 9 had a substance use disorder diagnosis.

**Table 1.** Characteristics of Study Participants at Baseline^a^

Characteristic	Mean (SD)	
	Control	Treatment
**Characteristics of patients**		
Total No.	1048	1187
No. of contactable practitioners		
All	1.64 (0.77)	1.64 (0.75)
Primary care managers	0.76 (0.43)	0.79 (0.41)
Opioid prescribers	0.74 (0.61)	0.71 (0.62)
Benzodiazepine prescribers	0.74 (0.53)	0.70 (0.53)
Prescribing during baseline period, d		
Opioid	31.0 (45.1)	30.7 (42.5)
Benzodiazepine	33.7 (34.4)	32.4 (33.6)
Opioid-benzodiazepine overlap	9.5 (15.2)	9.3 (15.2)
Age	47.9 (16.6)	48.7 (15.9)
Sex, No. (%)		
Female	596 (56.5)	679 (57.4)
Male	452 (43.5)	508 (42.6)
Mental health disorder diagnosis, No. (%)^b^		
Any diagnosis	548 (52.8)	640 (53.6)
Specified disorder		
Depressive	261 (25.3)	286 (23.8)
Anxiety/fear-related	361 (34.7)	418 (34.9)
Trauma/stressor-related	274 (26.7)	309 (25.7)
Posttraumatic stress	101 (10.0)	141 (11.5)
Suicidal ideation, No. (%)^b^	13 (1.3)	26 (2.2)
Substance use disorder diagnosis, No. (%)^b^	123 (11.9)	148 (12.3)
Opioid use disorder	40 (3.9)	39 (3.2)
**Characteristics of practitioners^c^**		
Total No.	325	429
Any patient in study in treatment group, No. (%)^d^	79 (27.5)	429 (100.0)
No. of patients in treatment group^d^	0.43 (0.86)	1.41 (0.93)
Prescribing during baseline period, d		
Opioid	186.6 (458.1)	210.5 (525.7)
Benzodiazepine	143.6 (296.0)	145.0 (345.2)
Opioid-benzodiazepine overlap	16.1 (35.1)	22.2 (69.3)
Sex, No. (%)		
Female	139 (43.7)	222 (51.0)
Male	186 (56.3)	207 (49.0)
Specialization, No. (%)		
Physician	259 (79.1)	331 (76.9)
Primary care physician	148 (45.7)	189 (43.8)
Psychiatrist or neurologist	21 (6.3)	33 (7.1)
Emergency medicine	16 (4.8)	31 (7.3)
Physician assistant	27 (8.5)	40 (9.3)
Nurse practitioner	19 (6.3)	35 (8.0)
Other	20 (6.1)	23 (5.9)

^a^ Values are means (SDs) weighted according to the inverse probability of treatment, except when otherwise noted that they are number of observations (% of observations); the % of observations is also weighted. All statistics on prescribing during the baseline period refer to the 90 days before the patient was enrolled (patient characteristics) or the 90 days before practitioner's first patient was enrolled in the study (practitioner characteristics).

^b^ Diagnoses from health care encounters during the year prior to the patient's enrollment.

^c^ Practitioners allocated to control or treatment group according to the assignment of their first patient enrolled in the study.

^d^ During the 90 days starting on the day the practitioner's first patient was enrolled.

These patients had 1830 practitioners when also counting those who worked outside the NCR but whose prescriptions were covered by the military health benefit. A total of 789 of these practitioners were in the NCR and thus eligible for emails and inclusion in practitioner-level analyses (eFigure 1 in Supplement 2). Based on the treatment allocation of their first patient enrolled in the study, 325 of these practitioners were in the control group for these analyses and 429 were in the treatment group. A total of 35 practitioners had multiple patients enrolled in their first week and were excluded.

As expected given the imperfect compliance design of the practitioner analyses, 27.5% of control practitioners had at least 1 patient in the treatment group in the subsequent 90 days (Table 1). Practitioners were mostly physicians, more than half of whom were primary care physicians. Nonphysician practitioners were mostly physician assistants and nurse practitioners.

### Patient-Level Outcomes

**Figure 2.**
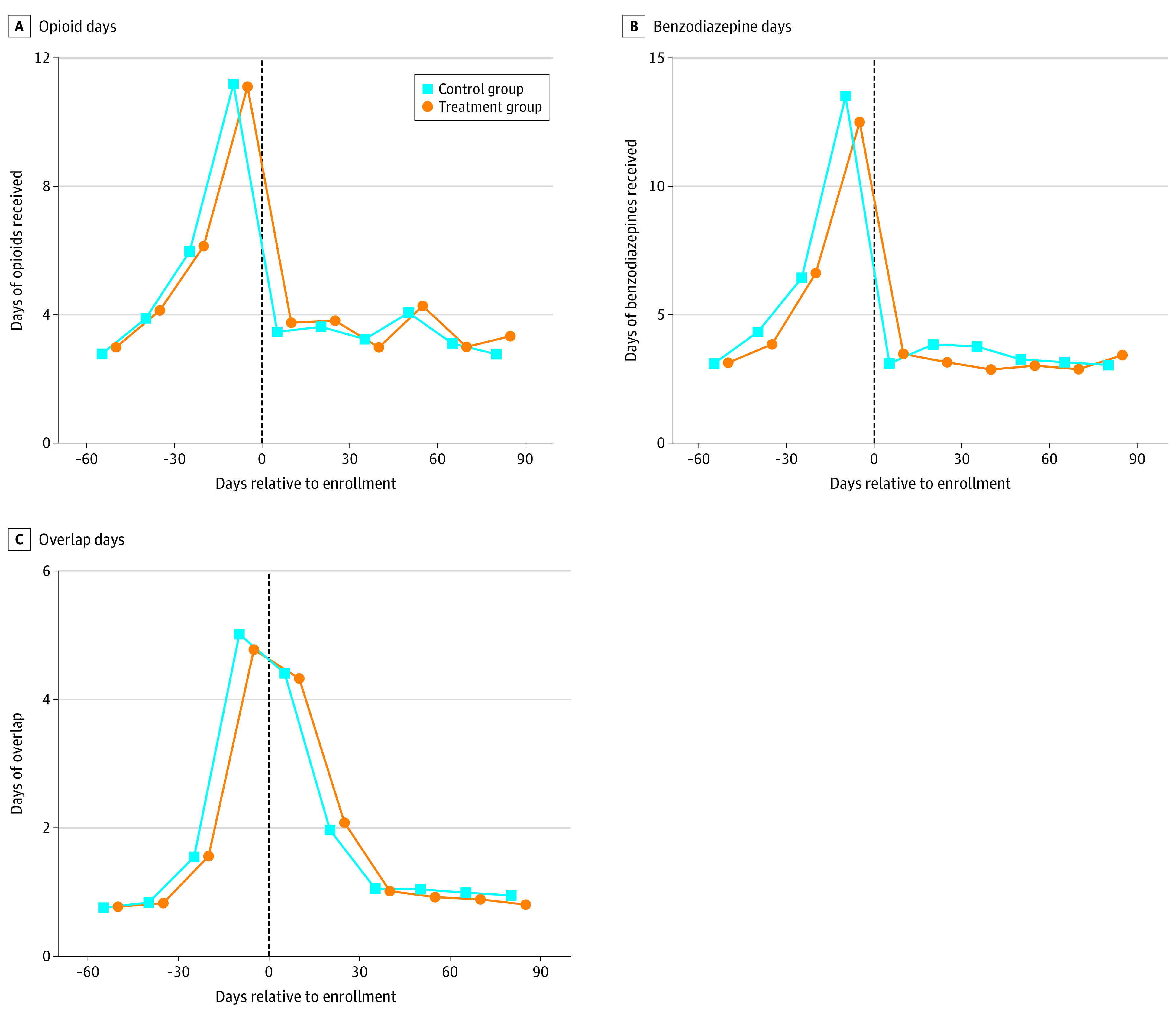
Receipt of Opioids and Benzodiazepines by Control and Treatment Group Patients Each point represents the mean days of drug receipt by patients in the control group and treatment group during a 15-day period relative to enrollment. Means use inverse probability of treatment weights based on the allocation ratio at the time of enrollment. Each panel considers the days of opioids, benzodiazepines, and overlapping opioids and benzodiazepines, respectively, the patient received during the period. Days of overlapping opioids and benzodiazepines calculated assuming patients start taking prescriptions on the day they fill them and continue taking them for the duration of their days' supply. The vertical line denotes the day of enrollment.

Following enrollment, patients’ mean receipt of opioids and benzodiazepines was similar in the control and treatment groups (Figure 2). Consistent with the visual evidence, there was no detected difference in opioid days (control mean: 20.2 days; treatment mean: 21.1 days, adjusted difference, 1.1 days; *P* = .81, 95% CI, -∞ to 3.0), benzodiazepine days (control mean: 20.2 days; treatment mean: 18.8 days, adjusted difference, −0.6 days, *P* = .30, 95% CI, -∞ to 1.4), or overlap days (control mean: 10.4 days; treatment mean: 10.0 days, adjusted difference, −0.1; days, *P* = .41, 95% CI, -∞ to 0.7) (Table 2). We also conducted a test of the joint hypothesis that effects on these endpoints were all 0. We failed to reject this joint hypothesis (*t* = .15; *P* = .56).

**Table 2.** Effect of Intervention on Primary and Key Secondary Outcomes^a^

Variable	Mean		Raw difference (95% CI)^b^	*P* value^c^	Adjusted difference (95% CI)^b^^,^^d^	*P* value^c^
	Control	Treatment				
**Patients (n = 2235)**						
Opioid days	20.2	21.1	0.9 (−∞ to 3.6)	.71	1.1 (−∞ to 3.0)	.81
Benzodiazepine days	20.2	18.8	−1.4 (−∞ to 1.0)	.17	−0.6 (−∞ to 1.4)	.30
Opioid-benzodiazepine overlap days	10.4	10.0	−0.4 (−∞ to 0.6)	.26	−0.1 (−∞ to 0.7)	.41
*P* value, all effects equal 0^e^			.37		.56	
**Practitioners (n = 754)**						
Opioid days	170.2	185.0	20.6 (−∞ to 89.4)	.69	−5.5 (−∞ to 15.7)	.34
Benzodiazepine days	128.6	134.8	6.1 (−∞ to 41.8)	.61	9.4 (−∞ to 26.6)	.82
Opioid-benzodiazepine overlap days	17.3	20.4	3.9 (−∞ to 9.4)	.88	0.0 (−∞ to 2.8)	.49
*P* value, all effects equal 0^e^			.72		.58	

^a^ All outcomes count prescribing during the 90 days after the patient was enrolled (patient outcomes) or the 90 days after the practitioner's first patient was enrolled in the study (practitioner outcomes) and are weighted according to the inverse probability of treatment.

^b^ One-sided 95% confidence interval.

^c^ These columns report *P* values from 1-sided tests (alternative hypothesis: effect <0) without accounting for multiple testing. eTable 2 in Supplement 2 presents multiple-testing adjusted *P* values.

^d^ Adjusted for opioid days, benzodiazepine days, and opioid-benzodiazepine overlap days during the baseline period to raise statistical power, as prespecified in the study analysis plan and described in main text.

^e^ To account for multiple testing, these rows report *P* values from joint 1-sided tests that the 3 above effects equal 0.

Results were similar at 30-day and 180-day durations as well as when measuring receipt in morphine or diazepam equivalent milligrams or number of fills. There were no detected impacts on receipt of naloxone, substance use disorder treatment medications, nonbenzodiazepine sleep medications, gabapentinoids, muscle relaxants, antipsychotics, or nonsteroidal anti-inflammatory drugs (eTable 3 in Supplement 2). Exploratory subgroup analyses by care team contact, including for patients whose entire care teams were contactable, also failed to detect any effects (eTable 4 in Supplement 2).

### Practitioner-Level Outcomes

**Figure 3.**
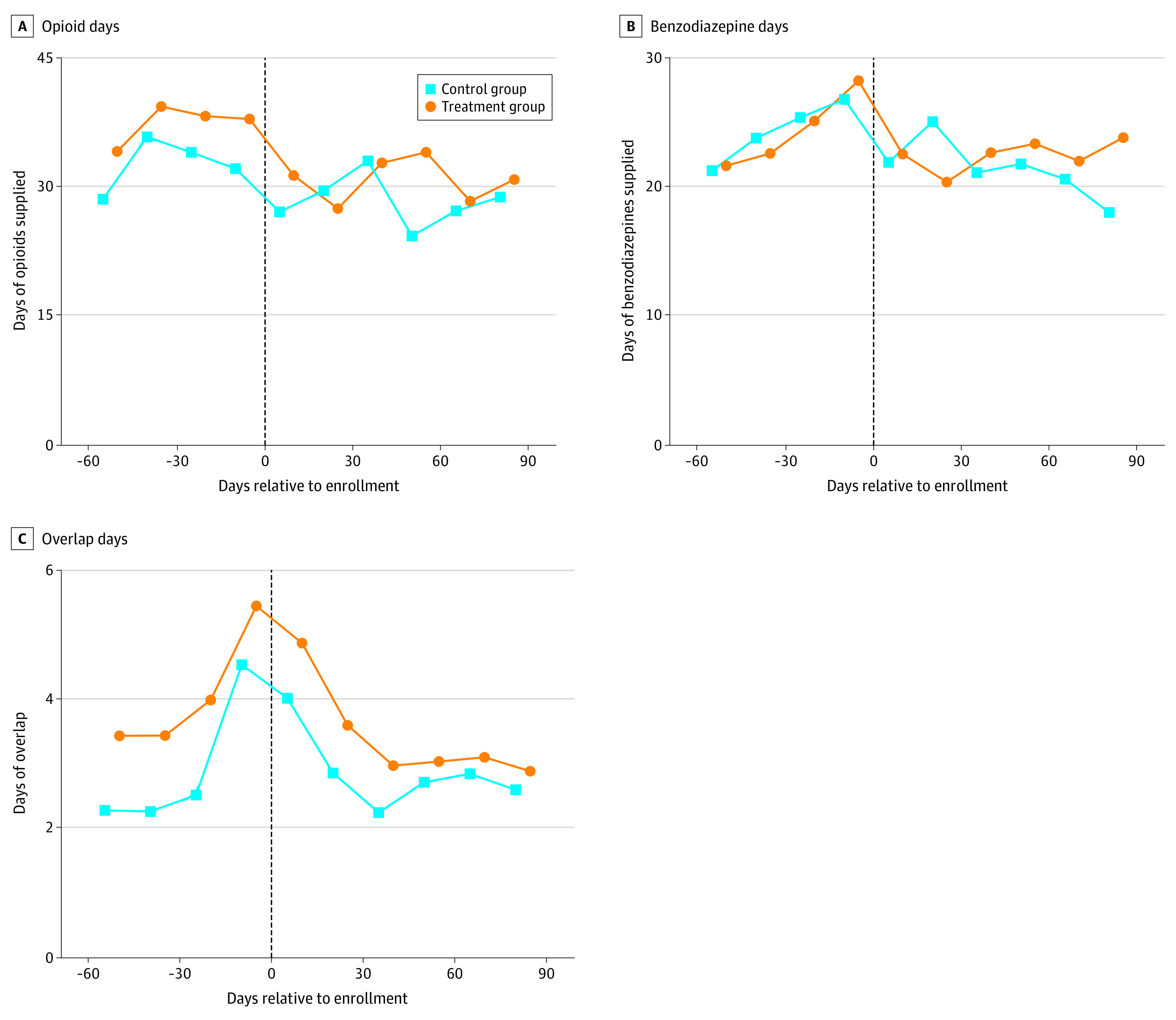
Prescribing of Opioids and Benzodiazepines by Control and Treatment Group Practitioners Each point represents the average days of drug supplied by practitioners in the control group and treatment group during a 15-day period relative to enrollment, defined as the date their first patient was enrolled in the study. Practitioners allocated to control or treatment group according to the assignment of their first patient enrolled in the study. Averages use inverse probability of treatment weights based on the allocation ratio at the time of enrollment. Each panel considers the days of opioids, benzodiazepines, and overlapping opioids and benzodiazepines, respectively, the practitioner supplied during the period. Overlapping opioid and benzodiazepine days defined as the number of patient-days to which the practitioner contributed. The vertical line denotes the day of enrollment.

Mean prescribing of opioids, benzodiazepines, and drugs in both classes together was similar for treated and control practitioners during the 90 days after their first patients were enrolled (Figure 3). We failed to detect differences in prescribing of opioids (control mean: 170.2 days, treatment mean: 185.0 days, adjusted difference, −5.5 days; *P* = .34, 95% CI, -∞ to 15.7), benzodiazepines (control mean: 128.6 days, treatment mean: 134.8 days, adjusted difference, 9.4 days; *P* = .82, 95% CI, -∞ to 26.6), or opioid-benzodiazepine overlaps (control mean: 17.3 days, treatment mean: 20.4 days, adjusted difference: 0.0 days; *P* = .49, 95% CI, -∞ to 2.8). We failed to reject a test of the joint hypothesis that effects on these endpoints were all 0 (*t* = .21; *P* = .58). We found similar results at shorter and longer durations and using alternative measures of prescribing volume and opioid-benzodiazepine overlaps. We also failed to detect any changes in prescribing of other relevant medications (eTable 5 in Supplement 2).

## Discussion

In this randomized clinical trial, sending email alerts to practitioners after their patients filled opioid and benzodiazepine prescriptions failed to detectably reduce patient receipt of these medications. These results are estimated precisely and we can rule out effects that are small but would have been clinically meaningful. For instance, using 2-sided tests we can reject that the intervention reduced patients’ receipt of opioids by more than 1.3 days, a 6.5% relative reduction compared with the control mean of 20.2 days.

We also hypothesized that the alerts would encourage practitioners to reduce coprescribing throughout their patient panels. If the alerts were particularly effective at curtailing new initiations of these medications, this effect could materialize even without detected effects on the patients mentioned in the emails. To test this hypothesis, we used the fact that a practitioner’s first patient enrolled in the study was randomly assigned to the treatment. However, this analysis also did not detect any prescribing effects.

These results contrast with prior successful opioid nudge interventions.^25,26,29^ However, interventions in this class are not universally effective. A recent systematic review of clinician nudges found that interventions that focused on providing or framing information often were not successful.^37^ Our findings also did not align with prior work suggesting that pharmacist interventions encourage safer prescribing. Given the earlier evidence and organizational experience suggesting their utility in encouraging safe prescribing,^38^ nudges engaging pharmacists in the care team remain a key topic for future research.

Our null findings demonstrate that combining QI activities with rigorous evaluation designs can produce valuable evidence for stakeholders. Traditionally, QI efforts have been evaluated with time series methods, but this approach can be at risk of confounding due to the phenomenon of regression to the mean.^39^ As an example, in the current study, practitioners in the control group prescribed approximately 10% fewer days of opioids and benzodiazepines in the 90 days after their first patient was enrolled compared with the 90 days before. A pre-post analysis would spuriously note benefits on these end points even though these prescribers received no email alert for that patient. Embedding randomization into our QI activities facilitated a rigorous evaluation with a null result. While the evaluation did not uncover a benefit of the intervention, it was still useful for the organization because it showed that limited health care resources could be more effectively deployed elsewhere.

This approach may also help organizations seeking to become learning health systems, defined by the Institute of Medicine as when “knowledge generation is so embedded into the core of the practice of medicine” that it “leads to continual improvement in care.”^40^ Randomized QI may help learning health systems identify practices with and without clinical benefits, facilitating a process of continuous improvement.^41,42,43^

### Limitations

This study has several limitations. We considered why these email alerts did not lead to detectable reductions in prescribing. First, there was a secular decline in coprescribing in the NCR in the leadup to this study. These successful efforts by stakeholders to reduce use of opioids and benzodiazepines may have limited the ability of our intervention to further change behavior. In addition, prescribers may have already integrated warnings about the dangers of coprescribing into their clinical practice,^14^ leaving less scope for this intervention to provide practice-changing information.^44^

Second, benefits of the intervention may have been too small or focused on a subset of patients to be detected. Most enrolled patients did not experience long-term coprescribing during which overdose risk and potential intervention benefits would have been highest. The alerts had limited potential benefits for patients who would not otherwise go on to fill more prescriptions, and several practitioners responded to the emails noting that they did not plan to continue prescribing. Alerts that occurred earlier in the prescribing process may have been more effective, as some research has suggested.^45^

Third, the alerts encouraged practitioners to coordinate with each other, and evidence suggests patients who receive coprescriptions from multiple practitioners or payors are at greater risk.^46,47^ While the message sought to promote safer prescribing through several channels, its ability to improve coordination was limited by the fact that 51% of study patients had one practitioner eligible for email. Efforts to ease coordination between practitioners separated geographically or across delivery systems may warrant further research; here, half of patients had practitioners who we could not contact because they worked outside the NCR or were community practitioners who could not be securely emailed.

Additional limitations include that actual enrollment was slightly below planned enrollment; that the treatment group includes the 140 patients randomized to similar email alerts in which practitioners were sent individual, not group messages; and that we could not ascertain whether practitioners opened the emails.

## Conclusions

The findings of this randomized clinical trial highlight the value of rigorous testing for health care organizations and policy makers. Ineffective interventions take staff away from valuable clinical activities and can even harm the efficacy of beneficial interventions through alert fatigue. Our results cast doubt on the utility of this intervention and others like it. By focusing on other avenues to make prescribing safer, organizations can avoid wasting scarce resources and creating unnecessary burdens for practitioners. The success of other interventions based on behavioral science suggests many opportunities remain for stakeholders seeking cost-effective approaches to promote safe prescribing of opioids, benzodiazepines, and other medications.

## Notes

Supplement 1.
Trial Protocol

Supplement 2. **eFigure 1.** CONSORT Flow Diagram of Practitioners in Study **eTable 1.** Additional Summary Statistics on Care Team Contact **eTable 2.** Comparison of P Values for Effect Estimates **eTable 3.** Effect of Intervention on Additional Patient-Level Outcomes **eTable 4.** Effect of Intervention on Patient Subgroups by Care Team Contact **eTable 5.** Effect of Intervention on Additional Practitioner-Level Outcomes

Supplement 3.
Data Sharing Statement
