# Supplementary material for: Effect of Pharmacist Email Alerts on Concurrent Prescribing of Opioids and Benzodiazepines by Prescribers and Primary Care Managers: A Randomized Clinical Trial
Source: JAMA Health Forum. 2022 Sep 30;3(9):e223378. doi: 10.1001/jamahealthforum.2022.3378 (PMC9526090; doi:10.1001/jamahealthforum.2022.3378)
Supplement: Supplement 1. — Trial Protocol [file jamahealthforum-e223378-s001.pdf]

Effect of Pharmacist E-mail Alerts on Concurrent Prescribing of Opioids and Benzodiazepines by Prescribers and Primary Care Managers: A Randomized Clinical Trial

Supplement 1: Study Methods and Analysis Plan

Adam Sacarny, PhD; Elana Safran, MPP; Mary Steffel, PhD; Jacob R. Dunham, MS; Orolo D. Abili, PharmD; Lobat Mohajeri, PharmD; Patricia T. Oh, PharmD; Alan Sim, PhD; Robert E. Brutcher, PharmD, PhD; Christopher Spevak, MD, MPH, JD

Table of Contents

STUDY METHODS 3
- **Intervention Design** 3
- **Evaluation Design** 4
- **References** 8
- **Intervention Samples** 10

ANALYSIS PLAN 13
- **Project Description** 13
- **Data and Data Structure** 13
- **Statistical Models & Hypothesis Tests** 18
- **Prescriber-Level Analysis** 19
- **References** 23
- **Updates and Deviations from Analysis Plan** 25

STUDY METHODS

The purpose of this study is to use informative e-mails to improve the process of prescribing of opioids and benzodiazepines within the National Capital Region/Military Health System (NCR/MHS), with the aim of decreasing concurrent opioid and benzodiazepine prescribing. Both the VA/DoD Clinical Practice Guideline for Opioid Therapy for Chronic Pain and the CDC Guideline for Prescribing Opioids for Chronic Pain strongly recommend against the concurrent use of opioids and benzodiazepines.[1–3] Taken together, these drugs could cause respiratory depression, enhanced sedation, and death.[4,5]

Intervention Design

Although there is a growing literature on interventions to improve guideline conformity of prescribing behavior, there is less evidence on stemming concurrent prescribing of opioids and benzodiazepines together. However, a growing literature shows that “nudges” and targeted communications to prescribers can be an effective way to improve guideline conformity of prescribing behavior.[6–9] These interventions can provide an opportunity to address common barriers to conformity with clinical practice guidelines, namely: knowledge of clinical guidelines, uncertainty about how to safely modify behavior to best align with guidelines, and coordination of care across multiple practitioners.[10–13]

Intervention

The intervention involved sending an e-mail alert to practitioners with information about the patient and an attached report detailing their concurrent prescriptions. Sample e-mails and reports are included in this supplement (Figures P1 and P2). The messages were sent over encrypted email (the NCR’s standard way to share patient information from the pharmacy department).

The **alert** (Figure P1) consisted of an encrypted email with the following practitioners copied together on one message: the patient's opioid prescriber(s), benzodiazepine prescriber(s), and/or primary care manager. The alert included relevant VA/DoD guidelines, stated the risk of concurrent prescribing to patient, and provided action steps and relevant resources. When multiple practitioners were identified, the message listed each of the practitioners’ contact information and explicitly encouraged them to coordinate with each other to revise the patient’s treatment plan to ensure their safety. When only one practitioner was identified, the message did not list contact information or include the line encouraging coordination.

*Initially, a **basic alert** approach was also tested. This approach differed in that the encrypted emails were sent separately to each of the practitioners, did not list practitioners’ contact information, and did not explicitly encourage the practitioners to coordinate. After 421 patients were enrolled (140 patients to the basic alert arm), the study team closed this arm to new enrollment. This decision was made in order to maximize the statistical power of the study to assess effects of the e-mail alert compared to no alert. The basic alert message matched what is depicted in Figure P1 except that the first bullet, which mentions coordination and lists practitioners’ contact information, was not included.*

During this time, the standard alert also matched what is depicted in Figure P1 except that above the numbered list it said, "Please take immediate action and coordinate with each other to revise your patient's treatment plan to ensure their safety." In addition, before July 29, 2019, the last line before the close read, "Thank you for taking immediate action and coordinating with each other to revise your patient's treatment plan to ensure their safety!"

During the initial period when the standard alert and basic alert were both being tested, study procedures differed slightly. We note the differences in italics in this supplement. In the analyses presented in the manuscript, we include patients in the basic alert arm. We do not distinguish between that arm and the standard alert arm, considering patients in both arms "treated".

The **report** (Figure P2) contained a summary of the patient's recent receipt of opioids and benzodiazepines as well as relevant information about risk factors and recent health care use related to overdose risk.

Evaluation Design

Treatment Conditions

The intervention was sent to the prescribers and primary care managers associated with patients who recently received concurrent prescriptions of opioids and benzodiazepines. Using a randomized approach, we allocated patients with concurrent prescriptions for opioids and benzodiazepines to two conditions:

1. E-mail Alert – The patient's opioid and benzodiazepine prescriber(s) and primary care manager all receive one e-mail alert in which they are copied together with the report attached.
2. As-Usual/Control - An as-usual approach, in which practitioners were not sent e-mails. These practitioners could access patient information through the MHS Opioid Registry as before.

Initially, a third condition was included. In the "basic e-mail alert" condition, the patient's opioid and benzodiazepine prescriber(s) and primary care manager each received a separate basic alert e-mail with the report attached. Study analyses presented in the manuscript retain patients randomized to the basic e-mail alert condition and consider those allocated both to the standard e-mail alert (#1 above) and the basic e-mail alert as "treated".

Enrollment

We enrolled patients who were identified as recently receiving overlapping opioids and benzodiazepines. These patients and their associated practitioners were identified using MHS administrative data and the MHS Population Health Portal Opioid Management Registry. We defined associated practitioners as the patient's PCM and any prescriber who wrote an opioid or benzodiazepine prescription that, according to its date of service and days' supply, ended within 30 days of the patient's last day of opioid-benzodiazepine overlap.

Practitioners outside the NCR were considered when calculating whether a patient experienced overlapping opioids and benzodiazepines but were not eligible to receive e-mails. We also did not contact PCMs if the patient did not have an appointment with them in the previous year.

The data was analyzed weekly to check for new patients who received concurrent opioids and benzodiazepines. These instances will be screened against the study exclusion and inclusion criteria.

Inclusion criteria were:

1. Patient had concurrent opioid-benzodiazepine prescriptions recently, defined as having an overlap day within the last 30 days at enrollment
2. At least one of the patient's practitioners (opioid prescribers, benzodiazepine prescribers, and/or PCM) was in the NCR and thus reachable by e-mail. The patient's PCM only counted for this determination if the patient had an appointment with them in the previous year. *Initially, this criterion was stricter: at least two of the patient's practitioners had to be in the NCR and thus reachable by e-mail.*

Prescribers were considered to be in the NCR if they had at least one appointment in the last month in an NCR facility. If a patient had a PCM on file, that PCM was considered to be in the NCR if the patient was enrolled in an NCR facility.

Exclusion criteria were:

1. Patient had a cancer diagnosis
2. Patient was receiving palliative care
3. Patient was under 18
4. Patient was previously enrolled

We also limited the number of new patients enrolled each week so that pharmacists were able to send all e-mails within the time allocated to the project. In initial enrollments in June 2019, the limit was 18 patients per week. The limit was adjusted over the following months to align with pharmacist resources. In December 2019 the limit was raised to 60 patients per week, where it remained for the rest of the study.

When there were more eligible patients than the enrollment limit, patients with the most recent final overlap day were enrolled first up until the limit. The remaining patients were not enrolled that week but remained eligible for enrollment in subsequent weeks.

Randomization / Assignment

The randomization was at the level of the patient. Each week, eligible patients were randomized at 1:1 ratio into the study conditions. Randomization was stratified based on each patient's number of opioid-benzodiazepine overlap days during the last 90 days at the time of enrollment. Specifically, we ordered the new patients by their number of overlap days and partitioned the set into blocks of 2. Each block therefore contained patients with consecutive values of overlap days. The randomization used a sequence of computer generated random numbers. The random numbers were stored on a separate computer system that was not accessible to the pharmacist team. Assignment to study arm using the random numbers was conducted automatically via SQL code.

Initially, patients were randomized at 1:1:1 ratio to the three conditions: basic e-mail alert, e-mail alert, and as-usual/control. Because there were three conditions, stratum were in blocks of 3. The main text considers patients allocated to both conditions as "treated". We therefore describe this initial period as randomizing at 2:1 ratio to the treatment and control groups.

Following enrollment each week, the pharmacist team downloaded a packet listing the patients enrolled into the treatment condition along with pre-populated e-mails and reports for their associated practitioners. Then, the pharmacists sent each patient's associated practitioners the e-mail alert(s) according to the pre-populated content with the reports attached.

Pharmacists were not blinded to study group assignments in that they observed patients and practitioners allocated to the treatment condition. Pharmacists were not provided with information on who was allocated to the control condition. *Since pharmacists sent the interventions, during the initial period when the basic alert condition was still open, they necessarily observed whether a practitioner was allocated to this condition or the regular alert.*

Power Calculation

The main text reports statistical power calculations and we provide additional details here. Using MHS data, we assembled a set of 2,286 pre-intervention patients in the NCR who received overlapping opioids and benzodiazepines during 2018Q2-2019Q1. We then assessed what the outcome variables for these patients would have been if the study been operating during this time. Assuming these patients would have been enrolled upon their first overlap day, we collected their days of opioids received, days of benzodiazepines received, and overlap days received during the following 90 days.

Because we planned to adjust study analyses for baseline measures of opioid and benzodiazepine receipt to raise statistical power, we also assembled these variables. Specifically, we collected patients' opioid days, benzodiazepine days, and overlap days received during the 30 days leading up to and including their first overlap day. We also collected baseline measures of opioid and benzodiazepine receipt in fills and morphine/diazepam milligram equivalents (MME/DME).

Next, we estimated the effective regression σ for each outcome. To do so, we regressed each outcome variable on the following baseline variables: opioid days, opioid fills, MME, benzodiazepine days, benzodiazepine fills, DME, opioid days * benzodiazepine days, opioid fills * benzodiazepine fills, MME * DME, and overlap days. Separate regressions were run for the subsample of patients with 1 NCR prescriber and the subsample of patients with 2 or more NCR prescribers. The residual variance estimates for each subsample were weighted by their shares of the total sample. We took the square root of this object, yielding the estimate of σ.

We then assessed minimum detectable effects (MDEs) using a two-sample means test assuming the variance was σ^2. The significance level was 5%, power was 80%, and tests were one-sided. The study size was assumed to be 2,500 patients. Summary statistics and the resulting power calculations are below. We report the mean and standard deviation of the given outcome variable, the estimate of σ, the effective R^2 (defined as $1 - [\sigma/\mathrm{SD}]^2$), and the MDE.

Outcome	Mean	SD	σ	Effective R^2	Study N	MDE
Days of Opioids	12.7	22.9	19.7	26.2%	2,500	2.0
Days of BZDs	11.8	22.2	18.9	27.8%	2,500	1.9
Overlap Days	3.8	11.8	11.0	12.0%	2,500	1.1

Ethics Approval

The intervention was conducted as a quality improvement project and was determined to be not human subjects research by the Walter Reed National Military Medical Center Department of Research Programs (Ref #910069, WRNMMC-EDO-2018-0246). The evaluation was conducted as human subjects research following approval by the Walter Reed National Military Medical Center Department of Research Programs Institutional Review Board (WRNMMC-EDO-2020-0452).

References

1. Rosenberg JM, Bilka BM, Wilson SM, Spevak C. Opioid Therapy for Chronic Pain: Overview of the 2017 US Department of Veterans Affairs and US Department of Defense Clinical Practice Guideline. *Pain Medicine*. 2018;19(5):928-941. doi:10.1093/pm/pnx203

2. Dowell D, Haegerich TM, Chou R. CDC Guideline for Prescribing Opioids for Chronic Pain — United States, 2016. *MMWR Recommendations and Reports*. 2016;65(1):1-49. doi:10.15585/mmwr.rr6501e1

3. Reisfield GM, Webster LR. Benzodiazepines in Long-Term Opioid Therapy. *Pain Med*. 2013;14(10):1441-1446. doi:10.1111/pme.12236

4. Turner BJ, Liang Y. Drug Overdose in a Retrospective Cohort with Non-Cancer Pain Treated with Opioids, Antidepressants, and/or Sedative-Hypnotics: Interactions with Mental Health Disorders. *J GEN INTERN MED*. 2015;30(8):1081-1096. doi:10.1007/s11606-015-3199-4

5. Park TW, Saitz R, Ganoczy D, Ilgen MA, Bohnert ASB. Benzodiazepine prescribing patterns and deaths from drug overdose among US veterans receiving opioid analgesics: case-cohort study. *BMJ*. 2015;350(jun10 9):h2698-h2698. doi:10.1136/bmj.h2698

6. Sacarny A, Barnett ML, Le J, Tetkoski F, Yokum D, Agrawal S. Peer Comparison Letters for High Volume Primary Care Prescribers of Quetiapine in Older and Disabled Adults: A Randomized Clinical Trial. *JAMA Psychiatry*. 2018;75(10):1003-1011. doi:10.1001/jamapsychiatry.2018.1867

7. Hallsworth M, Chadborn T, Sallis A, et al. Provision of social norm feedback to high prescribers of antibiotics in general practice: a pragmatic national randomised controlled trial. *The Lancet*. 2016;387(10029):1743-1752. doi:10.1016/S0140-6736(16)00215-4

8. Meeker D, Linder JA, Fox CR, et al. Effect of Behavioral Interventions on Inappropriate Antibiotic Prescribing Among Primary Care Practices: A Randomized Clinical Trial. *JAMA*. 2016;315(6):562. doi:10.1001/jama.2016.0275

9. Linder JA, Meeker D, Fox CR, et al. Effects of Behavioral Interventions on Inappropriate Antibiotic Prescribing in Primary Care 12 Months After Stopping Interventions. *JAMA*. 2017;318(14):1391. doi:10.1001/jama.2017.11152

10. Grol R, Wensing M. What drives change? Barriers to and incentives for achieving evidence-based practice. *Medical Journal of Australia*. 2004;180(S6). doi:10.5694/j.1326-5377.2004.tb05948.x

11. Grol R, Grimshaw J. From best evidence to best practice: effective implementation of change in patients' care. *The Lancet*. 2003;362(9391):1225-1230. doi:10.1016/S0140-6736(03)14546-1

12. Cabana MD, Rand CS, Powe NR, et al. Why Don’t Physicians Follow Clinical Practice Guidelines?: A Framework for Improvement. *JAMA*. 1999;282(15):1458. doi:10.1001/jama.282.15.1458

13. Davis D. The case for knowledge translation: shortening the journey from evidence to effect. *BMJ*. 2003;327(7405):33-35. doi:10.1136/bmj.327.7405.33

Intervention Samples

To: Practitioner A <practitioner.a@mail.mil>, Practitioner B <practitioner.b@mail.mil>
From: [redacted]@mail.mil
Subject: ** Immediate Action Needed: Patient At Risk **

Dear Dr. A and Dr. B,

Your patient, Jane Doe (EDIPN: XXXXXX), may be at risk for a life-threatening drug interaction between an opioid and a benzodiazepine. This information was obtained from a Carepoint MHS Population Health Portal opioid registry query (see attached report). The Defense Health Agency is taking steps to immediately alert prescribers and primary care managers (PCMs) when certain patients may be at risk.

Please take immediate action to ensure your patient's safety:
==

1. Please coordinate with your patient's care team to revise their treatment plan:

    -OPD/BZD Prescriber: Dr. A | NPI: 9999999
      practitioner.a@mail.mil | P:999-555-1111
    -PCM: Dr. B | NPI: 9999998
      practitioner.b@mail.mil | P:999-555-1113

2. Please assess the opioid treatment plan for your patient:
    - Consider tapering or discontinuing opioid (p. 13 https://[redacted])
    - Consider substituting treatments (https://[redacted])

3. Please assess the benzodiazepine treatment plan for your patient:
    - Consider tapering or discontinuing benzodiazepine (https://[redacted])
    - Consider substituting treatments (https://[redacted])

4. Please assess the risk management plan for your patient (pp. 46-51 https://[redacted]):
    - Consider overdose education and naloxone distribution (OEND)
    - Consider increased monitoring for overdose and suicidality

5. Please brief your patient on changes made:
    - Jane Doe | P:999-555-1222

==

The VA/DOD clinical practice guideline (CPG) for opioid therapy for chronic pain (pp. 43-44 https://[redacted]) and the BUMED INSTRUCTION 6320.101 about the Long-Term Opioid Treatment Program (p. 4 https://[redacted]) strongly recommend against the concurrent use of opioids and benzodiazepines. If taken together, they could cause respiratory depression, enhanced sedation, and death.

If you have questions about this message, email [redacted]@mail.mil. Thank you for taking immediate action to ensure their safety!

Sincerely,
LTC Robert E. Brutcher, PharmD, PhD
Department of Pharmacy

==

Confidentiality Notice:
This document may contain information covered under the Privacy Act, 5 USC 552(a), and/or the Health Insurance Portability and Accountability Act (PL 104-191) and its various implementing regulations and must be protected in accordance with those provisions. Healthcare information is personal and sensitive and must be treated accordingly. If this correspondence contains healthcare information it is being provided to you after appropriate authorization from the patient or under circumstances that don't require patient authorization. You, the recipient, are obligated to maintain it in a safe, secure and confidential manner. Re-disclosure without additional patient consent or as permitted by law is prohibited. Unauthorized re-disclosure or failure to maintain confidentiality subject you to application of appropriate sanction. If you have received this correspondence in error, please notify the sender at once and destroy any copies you have made.

Figure P1 – Sample E-Mail Alert Sent to Practitioners

Concurrent Opioid and Benzodiazepine Prescription Report

Patient Name: Jane Doe **PCM Name:** Practitioner B
DOB: 1-Jan-70 **GENDER:** F **BENCAT:** RTFMLY **EDIPN:** XXXXXX

Next Appt: 1-Jan-20 **Clinic:** Appt Clinic
Provider: Next Appt Provider **Appt Reason** Appt Reason

Overlapping Opioid and Benzodiazepine Prescriptions in Last 30 Days

Opioid Dispense Date	Opioid End Date	Opioid	MEDD	Benzo Dispense Date	Benzo End Date	Benzo
1-Jan-20	30-Jan-20	HYDROMORPHONE HCL (TABLET) 4MG #60 DS30 (A PHARMACY)	16	1-Jan-20	30-Jan-20	CLONAZEPAM #60 DS30 (A PHARMACY)

Medical History

Opioid Risk:
Active Opioid: YES **Active Benzo**: YES
Current Methadone Rx: NO **Current Long-Acting Opioid**: NO **Current Fentanyl Patch**: NO

Opioid Use Disorder Treatment/Prevention:
Active SL Buprenorphine Rx: YES **Active Naltrexone Rx**: NO **Last Naloxone Dispense Date**: 1-Jan-20

Healthcare Utilization:
ACG Emergency Visit Count: 0 **ACG Inpatient Hosp Count**: 0
ACG Major Procedure Flag: NO **SUICIDE:** NO **PALLIATIVE**: NO **HOSPICE**: NO

Mental Health Status (latest diagnosis date):
Depression: 1-Jan-20 **Other Mental Disorder**: 1-Jan-20 **Substance Use Disorder**:
PTSD: **Serious Mental Illness**:

Other Co-Morbidities (latest diagnosis date):
Obstructive Sleep Apnea: 1-Jan-20 **ACG Cancer Treatment (within the past 12 months):** NO

Figure P2.1 – Sample Report Attached to E-mail (Page 1 of PDF)

Report for: Jane Doe

Opioid Dispensings:

1-Jan-20	HYDROMORPHONE HCL (TABLET) 4MG #60 DS30 (A PHARMACY)	A, PRACTITIONER

Benzo Dispensings:

1-Jan-20	CLONAZEPAM #60 DS30 (A PHARMACY)	A, PRACTITIONER

Figure P2.2 – Sample Report Attached to E-mail (Page 2 of PDF)

Analysis Plan

This section repeats the study analysis plan that was archived on January 19, 2021, before the researchers were unblinded to post-intervention data. It makes only minor formatting changes to the original document. The plan refers to the Carepoint registry. We call this data source the MHS Population Health Portal Opioid Management Registry in the manuscript.

Project Name:
Reducing Concurrent Opioid-Benzodiazepine Prescriptions through Provider Messaging
Project Code: 1725
Date Updated: January 19, 2021

This document serves as a basis for distinguishing between planned (confirmatory) analysis and any unplanned (exploratory) analysis that might be conducted on project data. This is crucial to ensuring that results of statistical tests will be properly interpreted and reported. In order that the Analysis Plan fulfill this purpose, it is essential that it be finalized and date-stamped before we begin looking at the data — ideally, before we take possession of the data. Once this plan is finalized, a date is entered above, and the document is posted publicly on our team website.

Project Description

The purpose of this effort is to use low-cost informative e-mails to improve the process of prescribing of opioids and benzodiazepines within the National Capital Region/Military Health System (NCR/MHS), with the aim of decreasing concurrent opioid and benzodiazepine prescribing. Both the VA/DoD Clinical Practice Guideline for Opioid Therapy for Chronic Pain (2017) and the CDC Guideline for Prescribing Opioids for Chronic Pain (2016) strongly recommend against the concurrent use of opioids and benzodiazepines. Taken together, these drugs could cause respiratory depression, enhanced sedation, and death. The intervention population will be prescribers and primary care managers associated with patients who have recently received concurrent prescriptions of opioids and benzodiazepines. Using a randomized approach, we will allocate the NCR/MHS providers associated with patients with concurrent prescriptions for opioids and benzodiazepines to one of two conditions:

1. E-mail alert — A messaging approach, in which we will send encrypted emails to the patient's opioid and benzodiazepine prescriber(s) and primary care manager that identify the concurrent prescriptions and detail the patient's prescription history, inform them of the VA/DoD guideline and risk to patient, and provide action steps and relevant resources. When multiple providers are involved, the email message will also encourage coordination across providers and provide relevant contact information
2. As-Usual — An as-usual approach, in which providers are not sent messages. These providers can access patient information through the MHS Opioid Registry as before.

Data and Data Structure

This section describes variables that will be analyzed, as well as changes that will be made to the raw data with respect to data structure and variables.

Raw Data

Description	Summary of Data Fields	Purpose
Enrollment tracker data<br>(study enrollment table derived from Carepoint registry)<br>*Source: tracker and detail tables*	Patient ID<br>Provider ID<br>Treatment arm<br>Stratum<br>Enrollment date	Denominator of study population<br>Treatment assignment
Population summary data<br>(general information about the patients – one record for each patient in the study)<br>*Source: M2 (DEERS table), saved registry data*	Patient ID<br>Demographic information (age, race, sex)<br>Historical enrollment DMIS<br>Historical MHS coverage<br>Date of death	Baseline patient characteristics
Prescription fill data<br>(prescription drug fills associated with the patient population)<br>*Source: M2 (PDTS table), ESSENCE*	Patient ID<br>Date of service<br>Prescriber ID<br>NDC code<br>Days supply and quantity dispensed<br>*Patient and total dollars paid*	Prescribing outcomes
Professional provider encounter data<br>(professional encounters for the patient population)<br>*Source: M2 (CAPER, TED_NI tables)*	Patient ID<br>Date of service<br>Provider ID<br>Place of service<br>Type of service<br>Diagnosis codes<br>Procedure codes<br>*Patient and total dollars paid*	Professional provider utilization outcomes
Referral data<br>(patient referrals to other providers)<br>*Source: M2 (Referral table)*	Patient ID<br>Referral date<br>Referring provider ID<br>Type of service referred<br>Link to appointment/encounter record	Referral outcomes
Inpatient/ED provider encounter data<br>(facility encounters for the patient population)<br>*Source: M2 (CAPER, TED_NI, SIDR, TED_I tables)*	Patient ID<br>Date of service<br>Provider ID<br>Provider type<br>Diagnosis codes<br>Procedure codes<br>*Patient and total dollars paid*	Hospital utilization outcomes

Note: Referral data outcomes, as well as outcomes looking at patient and total dollars paid, are subject to availability of this data.

Outcome Variables to Be Analyzed

Primary Outcome

The primary outcome will consist of three components:

1. Opioid days
2. Benzodiazepine days
3. Days with overlapping opioids and benzodiazepines

Each component outcome will be measured at 90 days from the date the patient was enrolled. As we will describe later, the primary outcome will be adjusted for multiple testing.

Secondary Outcomes

We will also analyze a host of secondary outcomes. These outcomes fit into eight classes:

1. Robustness of primary outcomes to alternative definitions
    - Measuring each component at 30 days duration rather than 90 days
    - Measuring each component at 180 days duration rather than 90 days
    - Opioid MME (morphine milligram equivalent)
    - Benzodiazepine DME (diazepam milligram equivalent)
    - Opioid fills (rather than days supply of the fills)
    - Benzodiazepine fills (rather than days supply of the fills)
    - Indicator for having any opioid fill
    - Indicator for having any benzodiazepine fill
    - Days receiving over 90 MME/day of opioids
    - *Opioid cost ($)*
    - *Benzodiazepine cost ($)*
2. Prescribing of risk mitigation and use disorder treatment medications
    - Naloxone fills
    - Use disorder treatment medication (methadone, buprenorphine, naltrexone) days
3. Prescribing of alternative psychoactive medications
    - Non-BZD sleep aid days
    - Gabapentinoid days
    - Muscle relaxant days
    - Antipsychotic days
4. Prescribing of non-opioid pain relievers
    - NSAID days
5. Adverse changes in prescribing
    - Rapid opioid taper
    - Rapid benzodiazepine taper
6. Office visits with physicians, nurse practitioners, and physician assistants
    - Visits with the patient's prescriber(s) and/or PCM (as identified upon QI enrollment)
    - Visits with all physicians, nurse practitioners, and physician assistants
7. Use of pain management, mental, and behavioral health services
    - Visits with pain management physicians

- Visits to pain management specialists or clinics (including physicians)
- Visits with psychiatrists
- Visits to mental or behavioral health specialists or clinics (including psychiatrists)

8. *Referrals to pain management, mental, and behavioral health services*
    - *Referrals to pain management specialists or clinics (including physicians)*
    - *Referrals to mental or behavioral health specialists or clinics (including psychiatrists)*
9. Use of the hospital (inpatient and emergency department)
    - Visits to the hospital
    - Visits for mental health reasons
    - Visits for overdoses
    - Visits for use disorders

All prescribing and utilization outcomes will be created by transformations from the raw data described in the proceeding text. All measurements are at 90 days duration unless otherwise stated. *Analyses of referrals as well as opioid and benzodiazepine cost outcomes are subject to the availability of this data.*

Transformations of Variables

Prescribing outcomes

The primary outcomes will come from raw, prescription drug fill level data with dates of service during the outcome period. We will identify opioid fills by matching the National Drug Code (NDC) on the fill to the CDC opioid directory (https://www.cdc.gov/drugoverdose/resources/data.html). Opioid use disorder medications (methadone, buprenorphine, XR naltrexone) will not count as opioids, though we will study their receipt as a secondary outcome. We will identify fills for benzodiazepines and other medications using extracts from IBM Micromedex RED BOOK. When necessary, to account for new NDCs not yet included in the CDC directory or RED BOOK, we will use AHFS Therapeutic Class codes to identify opioids, benzodiazepines, and other medications.

To construct overlapping days of opioids and benzodiazepines, we will assume that each drug is active starting from its date of service and ending 'days supply'-1 days later. Then, we will calculate the number of days during which an opioid and a benzodiazepine were both active for the patient (Gellad et al. 2017, Sun et al. 2017).

Outcomes that state they will measure "days" will measure the "days supply" on prescription fills with dates of service during the outcome period. Outcomes that count fills will count the number of prescription drug fill records.

Outcomes using MME or DME will use equivalency tables to convert opioids and benzodiazepines of different strengths into common units: morphine equivalents for opioids and diazepam equivalents for benzodiazepines. The CDC data includes an equivalency table for opioids. Following other research on benzodiazepines, we will construct our own equivalency table for these drugs by referencing tables published in psychiatry, substance use, and addiction medicine textbooks. To ease the calculation of DME, we will only look at oral solids for this measure. For each prescription drug fill, we will multiply its number of units (i.e. number of

pills) by its strength per unit (available in the CDC data) and then by its MME/DME conversion factor to yield its MME/DME. Then we will sum the MME of all opioid fills to yield a total MME and we will sum the DME of the benzodiazepine fills to yield a total MDE.

To calculate the days the patient received more than 90 MME/day, we will assume each opioid fill is active from its date of service and ending 'days supply'-1 days later. We will equally apportion the total MMEs dispensed in the fill across the days. To determine if a patient received more than 90 MME on a day, we will sum the MME/day across all of their fills active on that day.

Rapid opioid and benzodiazepine tapers will be defined based on clinical practice guidelines and recent literature.

Utilization outcomes

Other utilization outcomes involve counting physician / nurse practitioner / physician assistant, clinic, inpatient, and ED visits or referrals. These outcomes will be constructed by processing raw encounter-level or referral-level data. Because single encounters may generate multiple claims or records in the data, the records will be aggregated to the patient-provider-day level before counting the number of visits. That is, multiple records for the same patient and provider in one day will not be counted as more than one visit. When encounters span multiple days (e.g. inpatient stays), they will be assumed to occur on the end date of service/discharge date.

Imported Variables

We will import data on pharmaceuticals from the CDC file: drug class, strength per unit, and MME conversion factor. We will construct our own table of DME conversion factors. When processing pharmaceuticals not included in the CDC file, we will use data from IBM Micromedex RED BOOK.

Transformations of Data Structure

The prescribing data will initially be at the fill level, i.e. each record will represent one dispense of a particular drug by a pharmacy to a patient. We will transform the data by collapsing it to the patient level, aggregating the dispensing events together to produce the three primary outcomes. We will then join the collapsed prescribing file with the enrollment file.

We will perform analogous transformations of the prescribing, encounter, and referral data to construct the secondary outcomes.

Data Exclusion

We do not anticipate making any data exclusions.

Treatment of Missing Data

Because we will use 100% administrative data, we do not anticipate any missing data. Prescribing outcomes will consider prescriptions filled through the Military Health System

(MHS), including direct care from MHS pharmacies and purchased care from non-MHS pharmacies.

Statistical Models & Hypothesis Tests

This section describes the statistical models and hypothesis tests that will make up the analysis — including any follow-ups on effects in the main statistical model and any exploratory analyses that can be anticipated prior to analysis.

Statistical Models

We will estimate the following two linear regressions:

$$y_i = \alpha + \beta^{y}_{raw} * T_i + Z_i\Theta + \varepsilon_i \text{ with weights } \omega_i\text{, and}$$

$$y_i = \alpha + \beta^{y}_{adj} * T_i + Z_i\Theta + X_i\Gamma + \varepsilon_i \text{ with weights } \omega_i,$$

where i indexes patients; y_i is the outcome (e.g. overlapping days, opioid days, etc.); T_i is an indicator for for assignment to the treatment arm; Z_i is a vector of indicators for strata, and X_i is a vector of pre-specified control variables. We use inverse probability of treatment weights (IPTWs) defined as:

$$\omega_i = T_i\left(\frac{T_{s(i)}}{N_{s(i)}}\right) + (1 - T_i)\left(\frac{\left(N_{s(i)} - T_{s(i)}\right)}{N_{s(i)}}\right),$$

where $s(i)$ is the stratum of patient i, T_s is the number of treated patients in stratum s, and N_s is the total number of patients in stratum s.

The former regression will yield "raw" or unadjusted estimates of the effect of the intervention. The latter regression will add control variables to raise statistical power and will produce adjusted estimates. All hypothesis tests for the primary outcomes will be based on the adjusted estimates.

The pre-specified control variables are:

- Lagged outcome i.e. the regression outcome defined over the time period immediately prior to study enrollment rather than after
- Lagged primary outcome components (opioid days, benzodiazepine days, and days with overlapping opioids and benzodiazepines) i.e. the vector of the three components defined over the 90 day period immediately prior to study enrollment rather than after
- Two-way interactions between the aforementioned variables

We will consider using an alternative machine learning approach to select the control variables.

Hypothesis Tests

To maximize statistical power, we will assess the primary outcome using one-sided tests to establish whether the treatment is superior to the control. We will report the p-value of the following joint test of the three primary outcome components:

$$H_0: \beta_{adj}^{opioids} = \beta_{adj}^{BZDs} = \beta_{adj}^{overlap} = 0$$

$$H_a: \beta_{adj}^{opioids} < 0 \; or \; \beta_{adj}^{BZDs} < 0 \; or \; \beta_{adj}^{overlap} < 0$$

In addition, to provide evidence on which endpoint, if any, was affected by the intervention, we will report for each: the point estimate, the p-value adjusted for multiple testing, and the p-value without adjustment for multiple testing. For each endpoint y we will test the following one-sided hypothesis:

$$H_0: \beta_{adj}^{y} = 0$$

$$H_a: \beta_{adj}^{y} < 0$$

Secondary outcomes will be assessed using two-sided tests:

$$H_0: \beta_{adj}^{y} = 0$$

$$H_a: \beta_{adj}^{y} \neq 0$$

Inference Criteria, Including Any Adjustments for Multiple Comparisons:

For inference, we will report design-based standard errors for all estimates of treatment effects. All joint tests will use the design-based variance matrix estimates. In this context, "design-based" refers to the standard errors that would describe how our treatment effect estimates would vary across multiple assignments of treatment following our design to the given experimental pool (i.e. robust standard errors).

For the primary outcome and primary outcome components, one-sided hypothesis tests with $P<0.05$ will be considered statistically significant. Multiple testing-adjusted p-values will be computed with the Westfall-Young algorithm.

The secondary endpoints will be assessed with two-sided hypothesis tests; $P<0.05$ will be considered statistically significant. These endpoints will be treated as exploratory and thus will not be adjusted for multiple testing.

Prescriber-Level Analysis

In addition, we will study the effects of the intervention at the prescriber level. These analyses are crucial to evaluating the intervention because it is possible for the intervention's effects to accrue not only to the patients mentioned in the e-mails but to providers' other patients as well.

For example, after receiving an e-mail, a provider may change their prescribing throughout their patient panel as they learn about the risks of opioid-benzodiazepine interactions.

Although the intervention was randomized at the patient level, it is possible to exploit this randomization to eliminate potential confounding in prescriber-level analyses. Our intuition is that in the first week a provider is enrolled in the study (i.e. the first week in which one of their patients is enrolled), the provider's assignment to the treatment or control arm is as good as random. After this week, whether the provider is subsequently enrolled and/or treated may no longer be random. Later enrollments reflect later prescribing behavior, and that behavior is potentially influenced by whether the provider was assigned to treatment during their first enrollment.

Our approach is to define providers as enrolling in the first week in which one of their patients was enrolled. If, during this first week, any of those patients were assigned to treatment, then the provider is considered treated; otherwise the provider is considered to be in the control group. We track their total prescribing over the following 90 days to construct study outcomes, as well as their total prescribing during the prior 90 days to construct baseline control variables. Then, we use a regression model to compare providers whose patients were randomized to treatment with providers whose patients were randomized to control.

Raw Data

These analyses will use the ***Enrollment tracker data*** and ***Prescription fill data*** described previously. The prescription fill data extract will consist of the same variables used in the patient-level analyses but will have a different definition of records: it will consist of all prescribing by study providers as well as all other prescriptions received by their patients, including prescriptions written by other providers. This extract will therefore allow us to measure total prescribing by providers in the study as well as opioid-benzodiazepine overlaps that involved prescribers outside the study.

Outcome Variables to Be Analyzed

Primary Outcome

The primary outcome of the prescriber-level analyses will be defined analogously to that of the patient-level analyses. It will consist of three components:

1. Opioid days
2. Benzodiazepine days
3. Days with overlapping opioids and benzodiazepines

Each component outcome will be measured at 90 days from the date the prescriber's first patient was enrolled. Effects on the primary outcome will be adjusted for multiple testing.

Secondary Outcomes

We will also analyze secondary outcomes. These outcomes come from the pre-specified patient-level secondary prescribing outcomes:

1. Robustness of primary outcomes to alternative definitions
   - Measuring each component at 30 days duration rather than 90 days
   - Measuring each component at 180 days duration rather than 90 days
   - Opioid MME (morphine milligram equivalent)
   - Benzodiazepine DME (diazepam milligram equivalent)
   - Opioid fills (rather than days supply of the fills)
   - Benzodiazepine fills (rather than days supply of the fills)
   - Indicator for having any opioid fill
   - Indicator for having any benzodiazepine fill
   - Patient-days receiving over 90 MME/day of opioids
2. Prescribing of risk mitigation and use disorder treatment medications
   - Naloxone fills
   - Use disorder treatment medication (methadone, buprenorphine, XR naltrexone) days
3. Prescribing of alternative psychoactive medications
   - Non-BZD sleep aid days
   - Gabapentinoid days
   - Muscle relaxant days
   - Antipsychotic days
4. Prescribing of non-opioid pain relievers
   - NSAID days

All measurements are at 90 days duration unless otherwise stated.

Transformations of Variables

See pre-specified patient-level analyses above.

Imported Variables

See pre-specified patient-level analyses above.

Transformations of Data Structure

We will transform the enrollment tracker data to be at the provider level. For each provider, we will capture its first week of enrollment, the number of times it was enrolled during that week, whether any of those enrollments were to the treatment arm, and whether the contact flag was on. Prescribing data will be transformed as described in the patient-level analyses, but will be collapsed to the provider-level rather than patient-level.

Data Exclusion

We anticipate excluding providers whose contact flag was off during the first week in which they were enrolled.

Treatment of Missing Data

See pre-specified patient-level analyses above.

Statistical Models

To derive our statistical model, first consider the following unadjusted regression:

$$y_i = \alpha + \beta^{y}_{unadj} * T_i + \varepsilon_i,$$

where i indexes providers; y_i is the outcome, and T_i is a binary indicator for whether any of the provider's patients enrolled during the first week were assigned to treatment. That is, if the provider had multiple patients enrolled during their first week, T_i indicates whether any of them were assigned to treatment.

There are two potential sources of confounding that could bias β^{y}_{unadj} in this regression. First, patients were enrolled to treatment vs. control at a 2:1 ratio early in the study and a 1:1 ratio later in the study. Thus providers who entered the study earlier had a higher chance of being assigned to treatment. We expect these providers to have higher propensities to prescribe, since they were flagged for the intervention earlier.

Second, providers with more patients flagged for enrollment during their first week are more likely to be assigned to treatment, since they have more draws in which they could be treated. We also expect these providers to have higher propensities to prescribe concurrently, since more of their patients were selected for the intervention.

The minimal specification must address these two potential sources of confounding. It does so by controlling for interactions between an indicator for the treatment vs. control enrollment ratio being 1:1 and a set of indicators for the number of times the provider was enrolled during their first week. We define this specification as the following linear regression:

$$y_i = \alpha + \beta^{y}_{raw} * T_i + Z_i\Theta + \varepsilon_i ,$$

where Z_i is the vector of interactions between enrollment ratio and the number of times the provider was enrolled during the first week.

We will also estimate a specification with additional adjustment:

$$y_i = \alpha + \beta^{y}_{adj} * T_i + Z_i\Theta + X_i\Gamma + \varepsilon_i,$$

where X_i is a vector of pre-specified control variables to raise statistical power. All hypothesis tests for the primary outcomes will be based on the adjusted estimates.

The pre-specified control variables are:

- A vector of indicators for the enrollment week
- Lagged outcome i.e. the regression outcome defined over the time period immediately prior to study enrollment rather than after
- Lagged primary outcome components (opioid days, benzodiazepine days, and days with overlapping opioids and benzodiazepines) i.e. the vector of the three components defined over the 90 day period immediately prior to study enrollment rather than after

- Two-way interactions between the aforementioned variables (other than the enrollment week indicators)

Hypothesis Tests

See pre-specified patient-level analyses above.

Inference Criteria, Including Any Adjustments for Multiple Comparisons

See pre-specified patient-level analyses above.

Exploratory Analysis

We expect to conduct an exploratory instrumental variables analysis at the prescriber level. Consider the following structural model:

$$y_i = \alpha^{SM} + \beta^{SM} * W_i + Z_i\Theta^{SM} + X_i\Gamma^{SM} + \varepsilon_i^{SM},$$

where W_i is an indicator for whether the prescriber was sent any e-mails during the 90 days following their first enrollment. Then β^{OLS} is the coefficient of interest, though an OLS estimate of the above will not yield the causal effect of being sent an e-mail on prescribing: first, there is reverse causation, since prescribing leads to further enrollments and further e-mails; second, there is confounding from the provider's reaction to their initial email, which could reduce prescribing and thus reduce the chance of being sent an e-mail in the future.

We instrument for W_i with T_i, the indicator for whether the prescriber was randomized to be sent an e-mail during their first week. The first stage is as follows:

$$W_i = \alpha^{1S} + \beta^{1S} * T_i + Z_i\Theta^{1S} + X_i\Gamma^{1S} + \varepsilon_i^{1S}.$$

We note that we expect the instrument to satisfy the three assumptions necessary for the above model to yield a causal interpretation. First, we know that conditional on the controls, T_i is assigned at random. Second, we know that T_i could only affect prescribing through the channel of causing an e-mail to be sent to the prescriber, W_i, satisfying the exclusion restriction. Third, we expect that being randomized to treatment raises the probability that the provider is ever e-mailed, satisfying the first stage requirement (we will be able to test whether there is a first stage in practice by estimating the above equation).

For completeness, we note that the reduced form equation matches our main prescriber-level specification:

$$y_i = \alpha^{RF} + \beta^{RF} * T_i + Z_i\Theta^{RF} + X_i\Gamma^{RF} + \varepsilon_i^{RF}.$$

<u>References</u>

Gellad WF, Zhao X, Thorpe CT, et al. Overlapping buprenorphine, opioid, and benzodiazepine prescriptions among Veterans dually enrolled in VA and Medicare Part D. *Substance abuse*. 2017;38(1):22-25. doi:10.1080/08897077.2016.1267071.

Sun EC, Dixit A, Humphreys K, Darnall BD, Baker LC, Mackey S. Association between concurrent use of prescription opioids and benzodiazepines and overdose: retrospective analysis. *The BMJ*. 2017;356:j760. doi:10.1136/bmj.j760.

<u>Updates and Deviations from Analysis Plan</u>

This section lists all differences between the analyses reported in this study and those that were proposed in the analysis plan.

Patient-Level Analysis

- We were not able to analyze secondary endpoints for opioid and benzodiazepine cost because this data was not made available to the research team.
- Because we failed to detect any changes in prescribing by any measure, we did not assess whether the intervention induced rapid opioid or benzodiazepine tapers. The binary outcomes 'receipt of any opioid' and 'receipt of any benzodiazepine' provide alternatives to these indicators, as well.
- Given the lack of changes in prescribing, the key target of the intervention, we also did not assess effects on health care utilization endpoints that would have been downstream of prescribing changes: practitioner office visits; use of pain management, mental, and behavioral health services; referrals to these services; or use of the hospital.
- eTable 4 in Supplement 2 presents a set of subgroup analyses by patients' care team contact. These analyses were not pre-specified.

Practitioner-Level Analysis

- The pre-specified analysis plan uses the term "provider" rather than "practitioner". To follow style guidelines, here and in the main text we use the latter term.
- The analysis plan refers to a set of "primary endpoints" for the practitioner-level analyses. However, it first defines a set of primary endpoints for the patient-level analysis. Because it is not standard practice to have two entirely separate sets of endpoints that are both considered primary, we refer to the practitioner-level endpoints as secondary in the main text.
- The analysis plan defines three practitioner-level primary endpoints analogous to the patient-level primary endpoints. We note our analysis plan document included tracked changes from February 2021 that limited the practitioner-level primary endpoint list to only one outcome, overlap days. These changes were not accepted prior to unblinding. Given the ambiguity of whether there are one or three endpoints, we opted to preserve the list before the (unaccepted) changes were added, which also has symmetry with the patient-level endpoints. Readers who would like to follow the approach in the tracked (but unaccepted) changes can consider the overlap days endpoint alone. Both approaches yield the same conclusion: that the e-mail intervention failed to detectably change prescribing.
- We use inverse probability of treatment weights (IPTWs) in all practitioner-level analyses, matching the patient-level approach. Each practitioner is given the IPTW of their first enrolled patient. Patients were initially enrolled at a 2:1 ratio to treatment or control before later switching to 1:1 ratio. Thus practitioners whose first patients were enrolled early were more likely to be treated than those whose first patients were enrolled later. This feature of the study would confound unweighted average differences between treatment and control practitioners. Including IPTWs addresses this issue and removes this confounding factor from the averages presented in Tables 1 and 2 and Figure 3 in the main text.
- Because we assign practitioners to treatment or control based on their first enrolled patient and randomization was at the patient-level, we cluster these analyses at the level of the first

enrolled patient. Thus practitioners with the same first enrolled patient are considered to be in the same cluster. Originally, the plan did not specify whether to cluster these analysis and if so, at what level.

- We originally planned to include practitioners who had multiple patients enrolled in the first week. However, as the "Statistical Models" section notes, including these practitioners would create an additional source of confounding because practitioners with more enrolled patients in the first week have a higher probability of being treated in that week and we expect that they are more likely to prescribe. To address this confounding, we would then have to augment the regression with indicators for the number of patients the practitioner had enrolled in their first week interacted with indicators for the allocation ratio in that week. Only 35 MHS practitioners (4% of the total) had more than 1 patient enrolled in their first week (eFigure 1 in Supplement 2). To avoid the complications to the statistical model that would be required to address confounding we opted to omit these practitioners. In turn, the sample consists of practitioners with just 1 patient enrolled in the first week. We do not include the indicator variable interactions because they would be fully absorbed by the enrollment week indicators.
- The analysis plan noted a potential exploratory instrumental variables (IV) analysis. The results we report in the main text would have been the reduced form to the IV. The IV estimates would have essentially scaled the reduced form estimates by first stage estimates. Because we failed to detect an effect in the main analyses, we did not pursue an IV approach.

Both Patient-Level and Practitioner-Level Analyses

- To simplify the statistical model, we did not include two-way interactions between the control variables as additional controls. We also did not pursue an alternative machine learning approach to select control variables.
- We anticipated reporting two p-values for each primary endpoint, one adjusted for multiple testing and another without adjustment. We pre-specified using the Westfall-Young algorithm for the adjustment. However, upon commencing analysis we learned that there is no established approach to using this algorithm in randomized experiments that are partially (but not entirely) paired, as this study is.
  Instead, the manuscript uses randomization inference and the Romano-Wolf stepdown procedure to adjust p-values for multiple testing. eTable 2 in Supplement 2 reports unadjusted analytic P-values, unadjusted randomization inference P-values, and Romano-Wolf adjusted P-values.
- We specified a joint hypothesis test with a null that effects on the three primary endpoints were all zero and a one-sided alternative that the effect on at least one endpoint was negative. When we commenced analysis, we learned that commonly used statistical packages like R, Stata, and SAS do not directly implement joint one-sided hypothesis testing. However, King and Smith (1986) propose a technically straightforward procedure for linear regression, and we use their approach.
  Specifically, we run a SUR that nests the three regressions used to estimate effects on the three endpoints. In this SUR, we constrain the treatment effect coefficients for each endpoint to equal each other:
  $$\beta^{opioids} = \beta^{BZDs} = \beta^{overlap} = \beta^{KS}$$
  and estimate β^{KS}. Then, we report the result from a one-sided t-test of

$$H_0: \beta^{KS} = 0 \text{ vs. } H_a: \beta^{KS} < 0$$

We also considered joint 2-sided tests using the same SUR approach without constraining that the treatment effect coefficients equal each other. We do not report P-values from these tests in the main text, but note that they failed to reject the null by wide margins for the patient-level and practitioner level endpoints.
