# Supplementary material for: Effect of Pharmacist Email Alerts on Concurrent Prescribing of Opioids and Benzodiazepines by Prescribers and Primary Care Managers: A Randomized Clinical Trial
Source: JAMA Health Forum. 2022 Sep 30;3(9):e223378. doi: 10.1001/jamahealthforum.2022.3378 (PMC9526090; doi:10.1001/jamahealthforum.2022.3378)
Supplement: Supplement 2. — eFigure 1. CONSORT Flow Diagram of Practitioners in Study eTable 1. Additional Summary Statistics on Care Team Contact eTable 2. Comparison of P Values for Effect Estimates eTable 3. Effect of Intervention on Additional Patient-Level Outcomes eTable 4. Effect of Intervention on Patient Subgroups by Care Team Contact eTable 5. Effect of Intervention on Additional Practitioner-Level Outcomes [file jamahealthforum-e223378-s002.pdf]

Supplemental Online Content

Sacarny A, Safran E, Steffel M, et al. Effect of pharmacist email alerts on concurrent prescribing of opioids and benzodiazepines by prescribers and primary care managers: a randomized clinical trial. *JAMA Health Forum.* 2022;3(9):e223378.
doi:10.1001/jamahealthforum.2022.3378

eFigure 1. CONSORT Flow Diagram of Practitioners in Study

eTable 1. Additional Summary Statistics on Care Team Contact

eTable 2. Comparison of P-Values for Effect Estimates.

eTable 3. Effect of Intervention on Additional Patient-Level Outcomes

eTable 4. Effect of Intervention on Patient Subgroups by Care Team Contact

eTable 5. Effect of Intervention on Additional Practitioner-Level Outcomes

This supplemental material has been provided by the authors to give readers additional information about their work.

© 2022 Sacarny A et al. *JAMA Health Forum*.

Online Figures

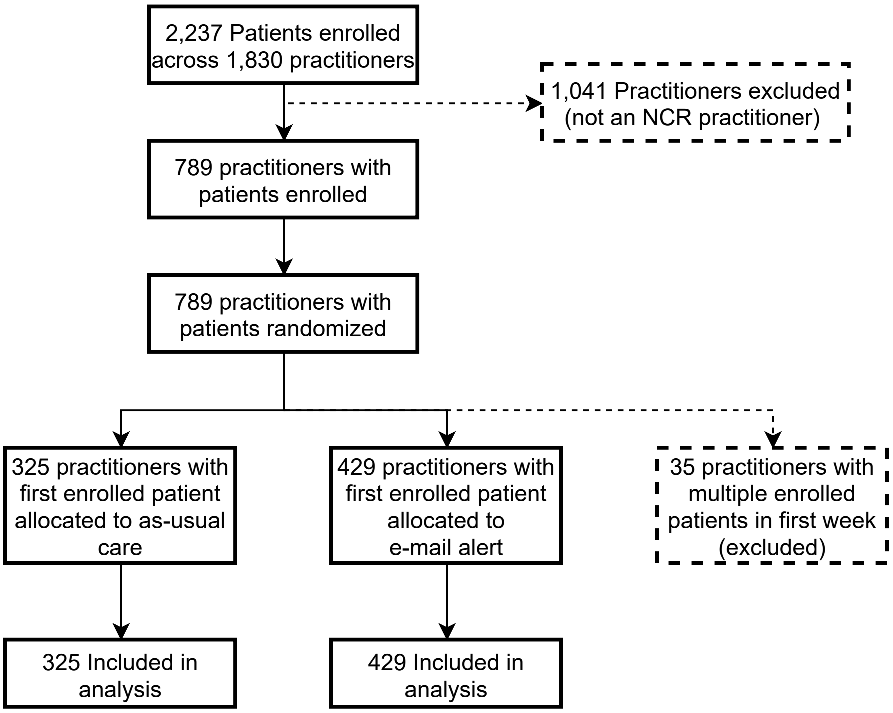

eFigure 1: CONSORT Flow Diagram of Practitioners in Study

© 2022 Sacarny A et al. *JAMA Health Forum*.

ONLINE TABLES

eTable 1: Additional Summary Statistics on Care Team Contact

Characteristics of Patients	Control (N=1,048)	Treatment (N=1,187)
At Least One Practitioner of Specified Type Contactable[a]		
Primary Care Manager	789 (76.3)	946 (79.0)
Who Was Also a Prescriber	213 (20.1)	236 (19.9)
Opioid Prescriber	681 (66.3)	765 (63.1)
Benzodiazepine Prescriber	714 (69.6)	812 (67.0)
Opioid/Benzodiazepine Prescriber	809 (78.6)	917 (75.8)
All Practitioners Contactable[a]	503 (48.9)	579 (47.6)
Opioid & Benzodiazepine Prescriber(s) Contactable[a]	586 (57.2)	660 (54.3)
Number of Contactable Practitioners (Repeated from Table 1)		
All	1.64±0.77	1.64±0.75
Primary Care Managers	0.76±0.43	0.79±0.41
Opioid Prescribers	0.74±0.61	0.71±0.62
Benzodiazepine Prescribers	0.74±0.53	0.70±0.53
Number of Non-Contactable Practitioners		
All	0.66±0.75	0.70±0.80
Primary Care Managers	0.07±0.25	0.07±0.25
Opioid Prescribers	0.45±0.60	0.47±0.62
Benzodiazepine Prescribers	0.33±0.49	0.36±0.53

* Plus-minus values are means ± standard deviations weighted according to the inverse probability of treatment.
a Number of observations (weighted percent of observations).

© 2022 Sacarny A et al. *JAMA Health Forum*.

eTable 2: Comparison of P-Values for Effect Estimates

	Estimated Treatment Effect (95% CI)[a,b]	Unadjusted Analytic P Value[c,d]	Unadjusted Rand. Inference P Value[c,e]	Multiple Testing Adjusted P Value[c,f]
Patients (N=2,235)				
Opioid Days	1.1 (-∞ to 3.0)	0.81	0.81	0.81
Benzodiazepine Days	-0.6 (-∞ to 1.4)	0.30	0.29	0.60
Opioid-Benzodiazepine Overlap Days	-0.1 (-∞ to 0.7)	0.41	0.38	0.60
Practitioners (N=754)				
Opioid Days	-5.5 (-∞ to 15.7)	0.34	0.36	0.65
Benzodiazepine Days	9.4 (-∞ to 26.6)	0.82	0.82	0.82
Opioid-Benzodiazepine Overlap Days	0.0 (-∞ to 2.8)	0.49	0.50	0.69

* All outcomes count prescribing during the 90 days after the patient was enrolled (patient outcomes) or the 90 days after the practitioner's first patient was enrolled in the study (practitioner outcomes) and are weighted according to the inverse probability of treatment.
a Analytic one-sided 95% confidence interval, not adjusted for multiple testing.
b Adjusted for opioid days, benzodiazepine days, and opioid-benzodiazepine overlap days during the baseline period to raise statistical power, as pre-specified in the study analysis plan and described in main text.
c These columns report p-values from one-sided tests (alternative hypothesis: effect<0).
d Analytic p-values without accounting for multiple testing. These values are reproduced from Table 2.
e Randomization inference p-values without accounting for multiple testing.
f Randomization inference p-values accounting for multiple testing using Romano-Wolf stepdown procedure. The three patient-level outcomes are treated as one family while the three practitioner-level outcomes are treated as another family.

© 2022 Sacarny A et al. *JAMA Health Forum*.

eTable 3: Effect of Intervention on Additional Patient-Level Outcomes

	Control Mean	Treatment Mean	Raw Difference (95% CI)[a]	P Value[b]	Adjusted Difference (95% CI)[a,c]	P Value[b]
Primary Outcomes, 30-Day Duration						
Opioid Days	7.1	7.5	0.5 (-1.1 to 2.0)	0.54	0.5 (-0.8 to 1.9)	0.44
Benzodiazepine Days	7.0	6.6	-0.3 (-1.7 to 1.1)	0.65	-0.1 (-1.4 to 1.2)	0.88
Opioid-Benzodiazepine Overlap Days	6.4	6.4	0.0 (-0.6 to 0.7)	0.92	0.2 (-0.4 to 0.8)	0.60
Primary Outcomes, 180-Day Duration						
Opioid Days	38.2	40.5	2.3 (-3.8 to 8.4)	0.46	2.6 (-1.7 to 6.9)	0.23
Benzodiazepine Days	37.5	35.6	-1.9 (-6.9 to 3.1)	0.46	-0.5 (-4.5 to 3.6)	0.82
Opioid-Benzodiazepine Overlap Days	15.5	14.6	-0.9 (-2.8 to 0.9)	0.33	-0.6 (-2.2 to 1.1)	0.50
Alternative Measures of Opioid and Benzodiazepine Receipt, 90-Day Duration						
Opioid mg (Morphine Equiv.)	708.6	874.8	166.2 (-62.9 to 395.3)	0.16	37.9 (-98.3 to 174.1)	0.59
Benzodiazepine mg (Diazepam Equiv.)	208.8	225.9	17.0 (-35.8 to 69.9)	0.53	-14.4 (-47.0 to 18.2)	0.39
Opioid Fills	1.0	1.0	0.0 (-0.1 to 0.1)	0.94	0.0 (-0.1 to 0.1)	0.79
Benzodiazepine Fills	0.7	0.6	0.0 (-0.1 to 0.0)	0.36	0.0 (-0.1 to 0.0)	0.40
Filled Any Opioid	38.1%	37.0%	-1.1pp (-4.7pp to 2.6pp)	0.57	-1.0pp (-4.4pp to 2.5pp)	0.57
Filled Any Benzodiazepine	33.3%	33.8%	0.4pp (-3.2pp to 4.1pp)	0.81	1.4pp (-1.9pp to 4.7pp)	0.41
Filled Opioid and Benzodiazepine	21.5%	19.0%	-2.5pp (-5.5pp to 0.5pp)	0.10	-2.1pp (-5.0pp to 0.8pp)	0.16
Opioid Days, >90 Morphine Equiv.	1.6	2.6	0.9 (0.0 to 1.9)	0.05	0.3 (-0.2 to 0.7)	0.28
Receipt of Other Medications, 90-Day Duration						
Naloxone Fills	0.0	0.0	0.0 (0.0 to 0.0)	0.26	0.0 (0.0 to 0.0)	0.25
Opioid Use Disorder Medication Days	0.5	0.9	0.4 (-0.2 to 1.0)	0.24	0.1 (-0.3 to 0.6)	0.55
Sleep Medication (ex. Benzo) Days	8.8	8.0	-0.8 (-3.2 to 1.6)	0.51	0.1 (-1.5 to 1.7)	0.87
Gabapentinoid Days	14.9	14.2	-0.7 (-3.7 to 2.4)	0.68	-1.3 (-3.7 to 1.1)	0.30
Muscle Relaxant Days	9.4	9.8	0.4 (-1.7 to 2.6)	0.71	0.0 (-1.7 to 1.7)	1.00
Antipsychotics Days	5.5	5.6	0.2 (-1.9 to 2.3)	0.86	-0.3 (-1.7 to 1.1)	0.68
NSAID Days	22.5	20.0	-2.6 (-6.0 to 0.9)	0.15	-2.8 (-5.9 to 0.3)	0.08

* All outcomes count prescribing during the 30, 90, or 180 days after the patient was enrolled as given in the heading and are weighted according to the inverse probability of treatment.

a Two-sided 95% confidence interval.

b These columns report p-values from two-sided tests (alternative hypothesis: effect≠0).

c Adjusted for opioid days, benzodiazepine days, and opioid-benzodiazepine overlap days during the baseline period to raise statistical power. When the outcome is not one of these three, the result is additionally adjusted for the given outcome measured during the baseline period.

© 2022 Sacarny A et al. *JAMA Health Forum*.

eTable 4: Effect of Intervention on Patient Subgroups by Care Team Contact

	Control Mean	Treatment Mean	Raw Difference (95% CI)[a]	P Value[b]	Adjusted Difference (95% CI)[a,c]	P Value[b]
A. Opioid or Benzodiazepine Prescriber Contactable (N=1,726)						
Opioid Days	20.4	21.3	0.8 (-∞ to 4.4)	0.65	1.7 (-∞ to 4.1)	0.86
Benzodiazepine Days	19.8	18.9	-0.9 (-∞ to 2.0)	0.31	-0.3 (-∞ to 2.1)	0.42
Opioid-Benzodiazepine Overlap Days	11.4	10.5	-0.9 (-∞ to 0.5)	0.14	-0.4 (-∞ to 0.6)	0.25
B. PCM Contactable and was a Prescriber (N=449)						
Opioid Days	32.1	32.8	0.7 (-∞ to 8.3)	0.56	3.0 (-∞ to 8.4)	0.82
Benzodiazepine Days	28.6	27.4	-1.2 (-∞ to 5.1)	0.37	4.4 (-∞ to 9.3)	0.93
Opioid-Benzodiazepine Overlap Days	14.6	12.4	-2.2 (-∞ to 1.2)	0.14	0.0 (-∞ to 2.5)	0.50
C. Opioid & Benzodiazepine Prescriber(s) Contactable (N=1,246)						
Opioid Days	16.6	19.1	2.4 (-∞ to 6.3)	0.85	1.5 (-∞ to 4.3)	0.81
Benzodiazepine Days	14.1	14.2	0.1 (-∞ to 3.1)	0.52	0.3 (-∞ to 2.7)	0.57
Opioid-Benzodiazepine Overlap Days	11.4	10.3	-1.1 (-∞ to 0.5)	0.13	-1.1 (-∞ to 0.1)	0.07
D. All Practitioners Contactable (N=1,082)						
Opioid Days	15.7	18.8	3.1 (-∞ to 7.2)	0.90	2.1 (-∞ to 4.9)	0.89
Benzodiazepine Days	15.0	14.8	-0.2 (-∞ to 3.1)	0.47	0.0 (-∞ to 2.8)	0.51
Opioid-Benzodiazepine Overlap Days	11.0	10.0	-1.0 (-∞ to 0.8)	0.18	-1.0 (-∞ to 0.3)	0.09

* This table presents exploratory (not pre-specified) subgroup analyses. All outcomes count prescribing during the 90 days after the patient was enrolled and are weighted according to the inverse probability of treatment. Because many strata would have only one observation in these analyses, they do not adjust for strata.
a One-sided 95% confidence interval.
b These columns report p-values from one-sided tests (alternative hypothesis: effect<0) without accounting for multiple testing.
c Adjusted for opioid days, benzodiazepine days, and opioid-benzodiazepine overlap days during the baseline period to raise statistical power.

© 2022 Sacarny A et al. *JAMA Health Forum*.

eTable 5: Effect of Intervention on Additional Practitioner-Level Outcomes

	Control Mean	Treatment Mean	Raw Difference (95% CI)[a]	P Value[b]	Adjusted Difference (95% CI)[a,c]	P Value[b]
Primary Outcomes, 30-Day Duration						
Opioid Days	56.7	58.9	3.6 (-22.0 to 29.2)	0.78	-3.5 (-14.0 to 6.9)	0.50
Benzodiazepine Days	47.0	42.9	-5.7 (-21.7 to 10.3)	0.48	-4.2 (-15.1 to 6.7)	0.45
Opioid-Benzodiazepine Overlap Days	6.9	8.5	1.8 (-0.8 to 4.3)	0.17	0.5 (-1.3 to 2.2)	0.60
Primary Outcomes, 180-Day Duration						
Opioid Days	315.1	341.3	36.3 (-106.7 to 179.3)	0.62	-11.3 (-64.2 to 41.6)	0.67
Benzodiazepine Days	241.1	257.4	16.6 (-59.7 to 93.0)	0.67	21.2 (-16.9 to 59.4)	0.27
Opioid-Benzodiazepine Overlap Days	29.4	35.0	7.6 (-3.9 to 19.2)	0.19	-0.2 (-5.6 to 5.2)	0.95
Alternative Measures of Opioid and Benzodiazepine Prescribing, 90-Day Duration						
Opioid mg (Morphine Equiv.)	5,309	4,918	-281 (-4,271 to 3,710)	0.89	-17 (-888 to 854)	0.97
Benzodiazepine mg (Diazepam Equiv.)	1,082	1,113	8 (-502 to 519)	0.97	106 (-56 to 269)	0.20
Opioid Fills	9.2	10.4	1.9 (-0.6 to 4.5)	0.13	0.6 (-0.7 to 2.0)	0.37
Benzodiazepine Fills	4.6	4.7	0.1 (-1.2 to 1.3)	0.92	0.2 (-0.5 to 0.8)	0.58
Prescribed Any Opioid	74.2%	74.1%	-0.1pp (-6.4pp to 6.3pp)	0.98	0.3pp (-5.1pp to 5.7pp)	0.91
Prescribed Any Benzodiazepine	64.1%	66.2%	4.1pp (-2.8pp to 10.9pp)	0.24	3.2pp (-3.1pp to 9.4pp)	0.32
Opioid Days, >90 Morphine Equiv.	10.7	9.3	-1.2 (-11.1 to 8.8)	0.82	-0.3 (-2.4 to 1.8)	0.79
Overlap Days, Prescribed Both[d]	6.4	8.2	2.2 (-1.7 to 6.2)	0.27	-0.1 (-2.8 to 2.6)	0.94
Overlap Days w/ Other Prescriber[e]	11.5	12.8	1.5 (-2.8 to 5.9)	0.49	-0.3 (-3.0 to 2.3)	0.80
Prescribing of Other Medications, 90-Day Duration						
Naloxone Fills	0.1	0.1	0.0 (-0.1 to 0.1)	0.90	0.0 (-0.1 to 0.1)	0.81
Opioid Use Disorder Medication Days	6.3	11.9	4.4 (-7.3 to 16.0)	0.46	4.7 (-4.4 to 13.8)	0.31
Sleep Medication (ex. Benzo) Days	199.0	214.0	11.4 (-54.7 to 77.6)	0.73	16.8 (-7.8 to 41.5)	0.18
Gabapentinoid Days	341.3	430.5	78.7 (-17.5 to 174.9)	0.11	13.7 (-26.0 to 53.4)	0.50
Muscle Relaxant Days	198.0	208.3	9.1 (-49.2 to 67.4)	0.76	-11.7 (-36.8 to 13.4)	0.36
Antipsychotics Days	72.4	65.9	-12.0 (-61.4 to 37.4)	0.63	7.1 (-7.3 to 21.6)	0.33
NSAID Days	2,060.3	2,193.0	178.1 (-350.9 to 707.1)	0.51	-91.1 (-230.7 to 48.5)	0.20

* All outcomes count prescribing during the 30, 90, or 180 days after the practitioner's first patient was enrolled in the study as given in the heading and are weighted according to the inverse probability of treatment.
a Two-sided 95% confidence interval.
b These columns report p-values from two-sided tests (alternative hypothesis: effect≠0).
c Adjusted for opioid days, benzodiazepine days, and opioid-benzodiazepine overlap days during the baseline period to raise statistical power. When the outcome is not one of these three, the result is additionally adjusted for the given outcome measured during the baseline period.
d Opioid-benzodiazepine overlap patient days in which the practitioner prescribed medications in both classes to the patient. Exploratory endpoint.
e Opioid-benzodiazepine overlap patient days in which the medications were prescribed by the practitioner and at least one other practitioner. Exploratory endpoint.

© 2022 Sacarny A et al. *JAMA Health Forum.*
