# Supplementary material for: Effect of Pharmacist Email Alerts on Concurrent Prescribing of Opioids and Benzodiazepines by Prescribers and Primary Care Managers: A Randomized Clinical Trial
Source: JAMA Health Forum. 2022 Sep 30;3(9):e223378. doi: 10.1001/jamahealthforum.2022.3378 (PMC9526090; doi:10.1001/jamahealthforum.2022.3378)
Supplement: Supplement 3. — Data Sharing Statement [file jamahealthforum-e223378-s003.pdf]

Data Sharing Statement

Sacarny. Effect of Pharmacist Email Alerts on Concurrent Prescribing of Opioids and Benzodiazepines by Prescribers and Primary Care Managers. *JAMA Health Forum*. Published September 30, 2022. doi:10.1001/jamahealthforum.2022.3378

Data
Data available: No

Additional Information
Explanation for why data not available: The data in this study consists of patient-level prescribing and enrollment information for people using the Military Health System, including servicemembers and their families. Due to the sensitive nature of this data, we cannot make it publicly available.
